# Temporal dynamics of the repeated bout effect on exercise-induced muscle damage: a systematic review and three-level meta-analysis

**DOI:** 10.3389/fphys.2026.1815013

**Published:** 2026-10-28

**Authors:** Fengyuan Yu, Lei Xu, Xingyi Su, Yijia Gao, Chen Chen, Laikang Yu

**Affiliations:** 1 P. E. Teaching Section, Changzhou Institute of Technology, Changzhou, China; 2 Department of Physical Education, Shijiazhuang University of Applied Technology, Shijiazhuang, China; 3 School of Martial Arts and Arts, Nanjing Sport Institute, Nanjing, China; 4 Beijing Key Laboratory of Sports Performance and Skill Assessment, Beijing Sport University, Beijing, China; 5 Department of Strength and Conditioning Assessment and Monitoring, Beijing Sport University, Beijing, China

**Keywords:** exercise-induced muscle damage, meta-analysis, preconditioning, repeated bout effect, time-course

## Abstract

**Objective:**

The repeated bout effect (RBE) confers protection against exercise-induced muscle damage (EIMD). However, the temporal profile of this protective response both across post-exercise recovery and across different intervention-to-damage intervals remains poorly defined. This three-level meta-analysis aimed to quantify the time-course of RBE protective effects and to characterize potential temporal windows for intervention and measurement.

**Methods:**

A systematic search of PubMed, Web of Science, Embase, and Scopus (inception to June 2025) identified 35 eligible studies. Three outcome measures, maximal voluntary contraction (MVC), delayed onset muscle soreness (DOMS), and creatine kinase (CK), were analyzed using three-level random-effects models. Effect sizes (Hedges’ g) were stratified by post-exercise measurement time (24, 48, 72, and 96 h) and intervention interval (<1 week, 1–2 weeks, 2–3 weeks, and >3 weeks).

**Results:**

RBE significantly protected MVC (overall g = 1.18, 95% CI: 0.91–1.45) and reduced DOMS (g = −1.37, 95% CI: −1.70 to −1.05) and CK (g = −0.91, 95% CI: −1.33 to −0.50). Time-stratified analyses revealed distinct temporal patterns. MVC and DOMS showed their largest observed protective estimates at 72 h post-damage (g = 1.47 and −1.65, respectively), whereas CK showed its largest observed protective estimate at 96 h (g = −1.53). Direct comparisons accounting for within-study dependence showed a larger MVC estimate at 72 h than at 48 h and a more protective CK estimate at 48 h than at 24 h. Subgroup analyses revealed outcome-specific interval patterns, with pooled protective effects for MVC and DOMS numerically smallest in magnitude in the 2–3-week subgroup at all four assessed time points.

**Conclusion:**

RBE-related protection showed time-dependent patterns across outcomes. Among the assessed time points, pooled protective effects were largest in magnitude at 72 h for functional (MVC) and perceptual (DOMS) outcomes, whereas biochemical (CK) effects increased progressively in magnitude through 96 h. The 2–3-week interval was characterized by numerically weaker pooled protective effects for MVC and DOMS. These findings provide an evidence-informed temporal framework for optimizing preconditioning and recovery-monitoring strategies in athletic training.

**Systematic review registration:**

https://www.crd.york.ac.uk/PROSPERO/, identifier CRD420251131290.

## 1 Introduction

Exercise-induced muscle damage (EIMD) is a common consequence of unaccustomed or high-intensity physical exertion, particularly that involving eccentric contractions. It manifests as reductions in maximal voluntary contraction (MVC), delayed onset muscle soreness (DOMS), restricted range of motion (ROM), and elevated serum biomarkers such as creatine kinase (CK) and myoglobin (MB) (Isner-Horobeti et al., 2013; Owens et al., 2019). These impairments can persist for several days and compromise subsequent training capacity. A well-documented adaptive phenomenon known as the repeated bout effect (RBE) confers partial protection against EIMD when a prior bout of exercise precedes the damaging stimulus. The mechanisms underlying the RBE are hypothesized to involve neural adaptations, modifications in fascicle and tendon unit behavior, and extracellular matrix remodeling (Hyldahl et al., 2017).

The RBE can be elicited through two general approaches. In the first approach, an identical high-intensity protocol is repeated, and the protective effect of this approach has been reported to last 2–8 weeks (McHugh and Tetro, 2003; Krentz and Farthing, 2010; Chen et al., 2012b; Munehiro et al., 2012; Doma et al., 2015; Doma et al., 2017). In the second approach, a low-volume or low-intensity preconditioning bout, typically 10%–40% of MVC, is administered 1 day to 2 weeks before the damaging exercise (Lavender and Nosaka, 2008; Chen et al., 2013b; Barreto et al., 2019). Both approaches have been shown to attenuate subsequent EIMD, though the extent and duration of protection vary considerably across studies depending on the interval between bouts, contraction type, exercise intensity, and the involved body region (Nosaka and Aoki, 2011).

To date, only one meta-analysis has systematically quantified the protective effects of preconditioning on EIMD. Boyd et al. (2023) focused primarily on low-intensity or low-volume preconditioning strategies and reported moderate protective effects across several EIMD markers. However, several important questions remain unaddressed. First, studies employing identical-intensity repeated bouts, which are arguably the most common paradigm in the RBE literature, were not the primary focus of that review. Second, and more critically, Boyd et al. (2023) pooled effect sizes across all post-exercise time points without examining how the protective effect evolves over the recovery period (e.g., at 24, 48, 72, and 96 hours post-EIMD). Third, the moderating role of the inter-bout interval, defined as the time elapsed between the conditioning bout and the damaging exercise, was not systematically evaluated across different interval durations. Given that the temporal characteristics of the RBE, including both the timing of the conditioning stimulus and the trajectory of recovery, are central to its practical application in training periodization, these gaps represent a meaningful limitation in the current evidence base.

To address these gaps, this study employs a three-level random-effects model that explicitly accounts for the statistical dependency among multiple effect sizes derived from the same study. This dependence is inherent in time-course data, in which each study contributes effect sizes at multiple post-exercise time points. This approach preserves all available time-point data while correctly partitioning variance into within-study and between-study components. The three outcome measures selected for analysis, MVC (muscle function), DOMS (subjective perception), and CK (biochemical marker), represent distinct physiological domains and enable examination of whether temporal patterns differ across measurement types.

## 2 Methods

This systematic review and meta-analysis followed the Preferred Reporting Items for Systematic Reviews and Meta-Analyses (PRISMA) guidelines for methodological and data reporting (Page et al., 2021). The Population, Intervention/Exposure, Comparison, and Outcome (PICO) framework was used to determine study eligibility and inclusion in this review. The protocol for this review has been registered in PROSPERO (registration number: CRD420251131290).

### 2.1 Inclusion and exclusion criteria

Studies meeting the following PICO criteria (**Table 1**) were included in this review. (1) Population: participants were healthy adults without serious underlying diseases or exercise-related disorders. (2) Intervention: the intervention involved the repeated bout effect (RBE), defined as the protective adaptation induced by a prior conditioning exercise bout before a subsequent muscle-damaging exercise bout. Eligible studies included between-participant comparator designs and within-participant, crossover, or repeated-bout designs evaluating RBE-related protection against exercise-induced muscle damage. (3) Comparator: When an explicitly defined no-RBE control group was available, it was used as the comparator. Eligible comparators performed the same or closely matched damaging exercise protocol as the RBE/preconditioning condition but did not receive a preceding conditioning stimulus. In studies without an explicit no-RBE control group, the comparator was defined according to the original study design as the bout or condition exposed to the damaging exercise without prior RBE/preconditioning exposure. These contrasts were considered internal/default comparators rather than randomized control comparisons. (4) Outcome measures: All included studies were required to report at least one of the following outcome measures: muscle strength function [e.g., maximal voluntary contraction (MVC), maximal voluntary isometric contraction (MVIC), maximal voluntary eccentric contraction (MVEC), and maximal voluntary concentric contraction (MVCC)]; muscle soreness [assessed using the Likert scale (Meneghel et al., 2014) or the visual analog scale]; muscle function [e.g., range of motion (ROM) and limb circumference]; and inflammatory blood biomarkers [e.g., creatine kinase (CK) and myoglobin (MB)]. All outcomes had to be assessed within 24, 48, 72, and 96 h following the muscle-damaging exercise bout. Studies were required to include at least two post-intervention assessments. Given the specificity of the intervention, only experimental studies were included, and all RBE/preconditioning conditions and corresponding comparator conditions were required to meet the above criteria.

**Table 1:** PICO framework.

PICO	Category
Population	Adults (18–60 years)
	Healthy; no injury at the intervention site and not recovering from other injuries or illnesses
Intervention	Resistance training, with the targeted muscle groups being homologous muscles
Control	Participants performed the same damaging exercise task as the RBE/preconditioning condition but without the preceding RBE stimulus. Studies without an explicitly designated no-RBE control group were not treated as randomized controls; their comparator conditions were coded as internal/default comparators and examined in sensitivity analyses.
Outcome	MVC, DOMS, CK

MVC, maximum voluntary contraction; DOMS, delayed onset muscle soreness; CK, creatine kinase.

Exclusion criteria were as follows: (1) Studies whose intervention protocols do not meet the criteria of this review (e.g., interventions with inappropriate intensity, methods, or forms for including the RBE). (2) Studies including participants with comorbidities or individuals in recovery (e.g., post-surgery or post-stroke recovery). (3) Studies reported only as abstracts, reviews, or case studies. (4) Studies published in languages other than English and without a provided translation.

### 2.2 Search strategy

Literature searches were conducted using the following databases: Web of Science, PubMed, Embase, and Scopus, with the search period spanning from database inception to June 29, 2025. Search terms included three categories: (1) preconditioning and repeated bout effect-related terms, (2) resistance training-related terms, and (3) muscle damage-related terms (**Supplementary Table S1**). Boolean operators (“AND”, “OR”) were used to combine keywords, including synonyms, antonyms, and common abbreviations. The search was not restricted by publication date. Eligible article types were RCTs, crossover studies, and English-language cross-sectional studies involving healthy adult human participants of all genders. Supplemental searches were conducted using reference lists from the included studies.

### 2.3 Study selection

Eligibility screening of the abstracts extracted from the literature search was performed independently by two researchers (FY and CC). Abstracts were categorized as either meeting the inclusion criteria (Yes) or not (No). Following abstract screening, the full texts of all eligible abstracts were further assessed using the same inclusion criteria. Any discrepancies in abstract screening and full-text assessment between the two authors were resolved by involving a third researcher (LX) until consensus was reached.

### 2.4 Data extraction

After literature screening, the following information was extracted from each included study: first author, publication year, participant characteristics, intervention details (interval time, targeted muscle groups, contraction type, contraction intensity), and outcome measure information (**Supplementary Table S2**). The mean values ± standard deviations (SD) of all outcome measures at 24, 48, 72, and 96 hours following the muscle-damaging exercise bout were recorded for the RBE/preconditioning condition and its corresponding comparator condition in a custom Excel sheet. For studies that reported outcome measures graphically, corresponding authors were contacted via email to request the original data. When no response was received from corresponding authors, the Web Plot Digitizer software (Copyright 2010–2024 Ankit Rohatgi) was used to extract data from figures. Studies from which data could not be extracted were excluded from the analysis. Unique participants refer to distinct individuals contributing data to the included analyses. Condition observations refer to participant contributions to distinct exercise conditions, with each individual counted once per condition. In within-participant designs, the same individual may contribute to both the RBE/preconditioning and comparator conditions. Repeated assessment times do not increase either count. For paired comparisons, the analytical sample size is the number of individuals with data available for both conditions. Outcome-specific participant counts may overlap and should not be summed across outcomes.

### 2.5 Methodological quality assessment

The methodological quality of each included study was assessed using the Kmet evaluation checklist for quantitative research. The Kmet scoring tool uses a three-point ordinal scale (“Yes” = 2, “Partial” = 1, “No” = 0) and has demonstrated established validity and reproducibility (Kmet et al., 2004). Given that several Kmet criteria were not applicable to the diverse study designs included in this study, the following criteria were modified: Criterion 4 [Are the characteristics or input variables/information of the participants (and the control group, if applicable) adequately described, such as for decision analysis]?; Criterion 5 (Is random assignment to the treatment group described, if applicable)?; Criterion 6 (Is blinding of the intervention and researcher to the intervention reported, if possible)?; Criterion 7 (Is blinding of the intervention and participants to the intervention reported, if possible)?; Criterion 12 (Is confounding controlled)? (Boyd et al., 2023). Individual study quality scores were categorized using the following equation: A = Excellent (>23), B = Good (>18), C = Fair (>14), or D = Poor (<14) (Kmet et al., 2004). Two reviewers (FY and CC) independently assessed the quality and risk of bias of the included studies. In cases of discrepancy, consensus was first sought through discussion. If disagreements persisted, a third reviewer (XS) reviewed the relevant literature, and the final decision was made based on the majority opinion following joint review by all three reviewers.

The two authors (FY and CC) used the Grading of Recommendations, Assessment, Development, and Evaluation (GRADE) protocol to assess the certainty of evidence for each outcome, with ratings ranging from “very low” to “high” (Guyatt et al., 2011). Evidence could be downgraded due to the following factors: (1) Risk of bias: Evidence was downgraded by one level if the study’s Kmet score was fair (<67%) and by two levels if the Kmet <50%; (2) Indirectness: Evidence was downgraded by one level if the study population included individuals who had undergone prior training, even if they met the eligibility criteria, to ensure the specificity of the intervention and outcomes; (3) Publication bias risk: Evidence was downgraded by one level if publication bias was suspected; (4) Inconsistency: Evidence was downgraded by one level between-study heterogeneity (*I*^2^ value) exceeded 75%; (5) Imprecision: Evidence was downgraded by one level if the total number of participants across studies was fewer than 800.

### 2.6 Statistical analysis

Hedges’ g was calculated in StataMP with a small-sample correction. Outcome values were extracted in the form reported by each primary study: absolute post-exercise values, values normalized to each bout’s own baseline, or changes from baseline. The corresponding RBE/preconditioning and comparator values were compared at the same post-exercise assessment time. For between-participant comparisons, sampling variances were estimated using the independent-groups formulation. For within-participant, crossover, or repeated-bout designs, sampling variances were recalculated under assumed within-participant correlations of r = 0.30, 0.50, and 0.70, because most primary studies did not report the correlations required for exact paired-effect variance estimation. The computed Hedges’ g values and their corresponding sampling variances were then imported into R software (version 4.1.2) and analyzed using the metafor package (version 3.0-2) for multilevel meta-analysis. Because several studies contributed more than one effect size through multiple intervention groups, repeated-measures or crossover designs, multiple outcome measures, or repeated post-exercise time points, the primary analysis used a three-level random-effects model implemented with rma.mv in the metafor package. This model distinguishes three sources of variance: sampling error (Level 1), effect-size variation within the same study (Level 2), and between-study variability (Level 3) (Cheung, 2019). Models were fitted using restricted maximum likelihood estimation, and pooled effects were reported as Hedges’ g with 95% confidence intervals and prediction intervals. As a sensitivity analysis, corresponding two-level random-effects models were fitted using rma.uni to examine whether the pooled estimates were materially altered by the multilevel specification.

Influence diagnostics, including standardized residuals, leverage values, and Cook’s distance, were used to identify potentially influential studies. Models were then refitted after excluding these studies to assess the stability of the pooled estimates. For heterogeneity assessment, likelihood-ratio tests were used to sequentially fix the Level 2 or Level 3 variance terms and determine the significance of variability at each level. Furthermore, following the method proposed by Cheung (2019), the proportion of total variability contributed by each of the three levels (Level 1, 2, and 3) was calculated (*I*^2^).

In moderator analysis, a multilevel meta-regression model was constructed to sequentially test the impact of potential moderators (e.g., body region, intervention type, contraction type, contraction intensity, interval time, and measurement time) on effect size, using alternative reference categories. Continuous variables (e.g., measurement time) were modeled using linear regression, and significant variables were included in a multivariable regression model to compare the explanatory power of different combinations of variables.

Publication bias was explored using Egger’s regression test and funnel plots based on the two-level model to examine the systematic relationship between study precision and effect size. Additionally, a precision metric (SDA = $\sqrt{\frac{4}{N}}$) was constructed to further assess bias trends using weighted regression within the three-level structure. The Trim-and-Fill method was used as an exploratory sensitivity analysis to assess the potential impact of missing studies on the pooled effect estimate.

To examine the temporal profile of RBE-induced protection, separate three-level meta-analyses were conducted for each combination of outcome measure (MVC, DOMS, CK) and measurement time point (24, 48, 72, and 96 h post-damage exercise), yielding 12 time-specific effect estimates. Time-point differences were estimated by combining the four time-specific estimators for each outcome in a block-diagonal working model, with their separately REML-estimated Level 2 and Level 3 variances. Inference used study-clustered CR2 covariances and Satterthwaite degrees of freedom. Holm adjustment covered all six pairwise time comparisons within each outcome. Sensitivity analyses restricted the data to studies reporting all four time points. Secondary linear and quadratic time meta-regressions used CR2 inference, with Holm adjustment across the three quadratic terms. Time contrasts, time meta-regressions and interval contrasts were analyzed in R (version 4.5.1), using metafor (version 5.2-1) and clubSandwich (version 0.7.0). Separate three-level meta-analyses were conducted for each interval–measurement-time combination, yielding 48 pooled effects. Secondary interval contrasts were estimated using three-level REML meta-regression with categorical interval and measurement time as additive predictors and random intercepts for studies and effect sizes. Sampling variances formed a diagonal working covariance matrix. Inference used study-clustered CR2 covariances and Satterthwaite degrees of freedom. Holm adjustment covered the two contrasts (2–3 versus 1–2 and >3 weeks) within each outcome and analysis dataset. Sensitivity analyses additionally adjusted for upper- versus lower-limb region or restricted the data to eccentric preconditioning.

To evaluate whether methodological classification influenced the pooled estimates, each effect size was coded by study design (between-participant/control-comparator vs within-participant/crossover/repeated-bout) and comparator definition (explicitly specified control/comparator vs internal/default comparator). Studies contributing more than one comparison structure were coded at the effect-size level. For within-participant or crossover comparisons, sampling variances were recalculated under assumed within-participant correlations of r = 0.30, 0.50, and 0.70. Separate stratified random-effects sensitivity analyses were conducted by study design and comparator definition. These analyses were interpreted as robustness checks rather than replacements for the primary three-level model.

## 3 Results

### 3.1 Study selection process

A total of 17,133 records were retrieved from four databases. After 9,295 duplicate records were removed, 7,838 records were screened. Based on the predefined inclusion criteria, 35 studies were included in the systematic review and meta-analysis (**Figure 1**).

**Figure 1:**
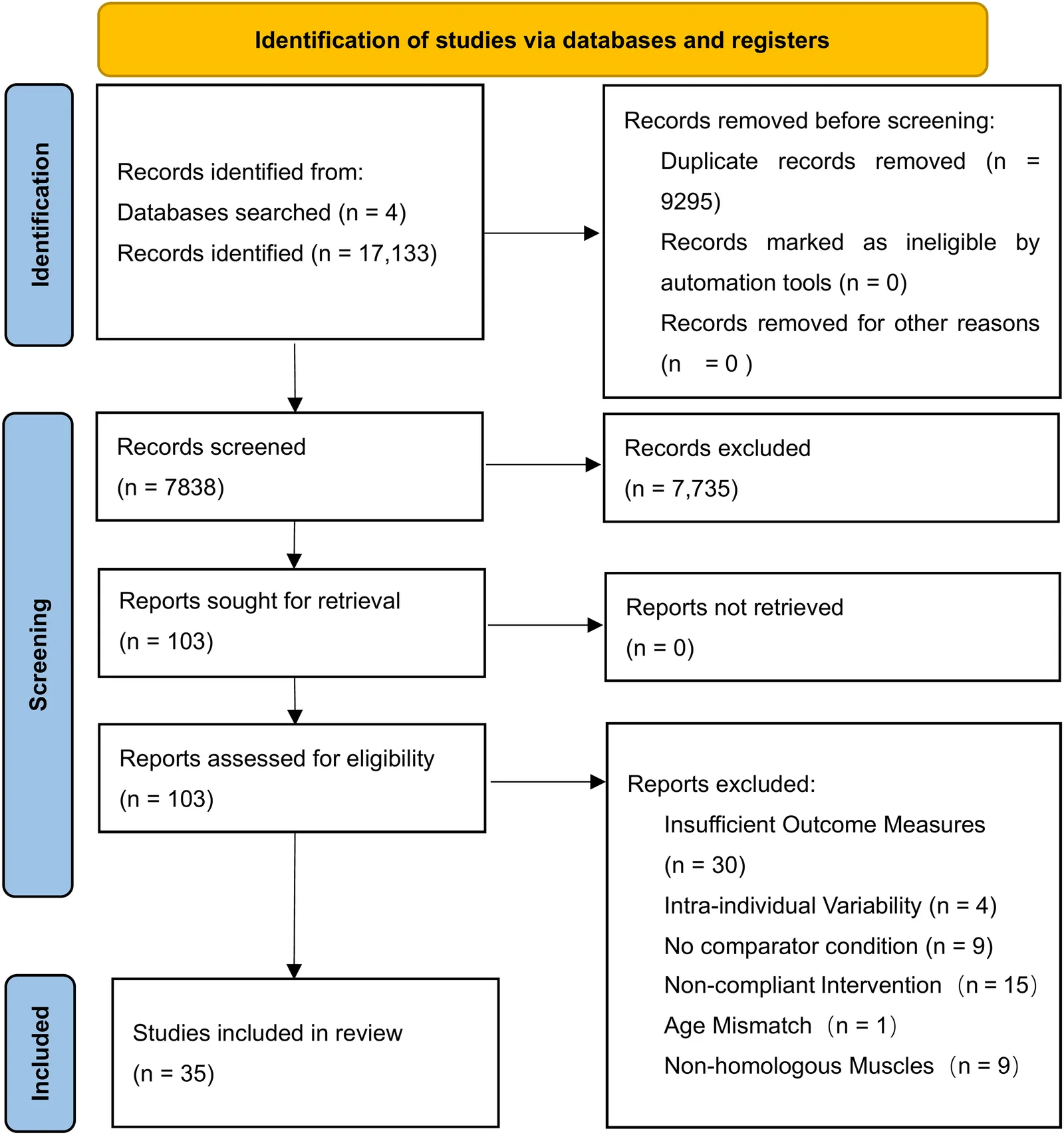
Selection process and search strategy for studies included in the systematic review and meta-analysis.

### 3.2 Characteristics of included studies

Thirty-five studies were included in this review. Study-specific sample sizes and participant characteristics are summarized in **Supplementary Tables S2A** and **S3**. In within-participant designs, the same participants contributed to both the RBE/preconditioning and comparator conditions. Consequently, condition-specific counts are not mutually exclusive and should not be added to obtain the total number of unique participants.

At the study level, 18 studies (Smith et al., 1994; Chen and Hsieh, 2000; Nosaka et al., 2001; Paddon-Jones and Abernethy, 2001; Chen, 2003; Chen and Nosaka, 2006; Chen et al., 2007; Lavender and Nosaka, 2008; Chapman et al., 2011; Chen et al., 2012b; Chen et al., 2012a; Chen et al., 2012; Chen et al., 2013a; Lin et al., 2015; Tseng et al., 2016; Barreto et al., 2019; Huang et al., 2019; Ho et al., 2021) provided only between-participant/control-comparator evidence, 15 studies (Ebbeling and Clarkson, 1990; Nosaka et al., 1991; Nosaka and Clarkson, 1997; Foley et al., 1999; Howatson et al., 2007; Nikolaidis et al., 2007; Chen et al., 2009; Barroso et al., 2010; Janecki et al., 2011; Muthalib et al., 2011; Meneghel et al., 2014; Lau et al., 2015a; Zourdos et al., 2015; Hinks et al., 2021; Chen et al., 2023) provided only within-participant/crossover/repeated-bout evidence, and two studies (Brown et al., 1997; Nosaka et al., 2005a) contributed both structures. At the effect-size level, 468 effect sizes were classified as between-participant/control-comparator comparisons and 204 as within-participant/crossover/repeated-bout comparisons.

With respect to comparator definition, 17 studies (Smith et al., 1994; Chen and Hsieh, 2000; Nosaka et al., 2001; Paddon-Jones and Abernethy, 2001; Chen, 2003; Chen and Nosaka, 2006; Lavender and Nosaka, 2008; Chapman et al., 2011; Chen et al., 2012b; Chen et al., 2012a; Chen et al., 2012; Chen et al., 2013a; Lin et al., 2015; Tseng et al., 2016; Barreto et al., 2019; Huang et al., 2019; Ho et al., 2021) used an explicitly specified no-RBE control/comparator condition, whereas 18 studies (Ebbeling and Clarkson, 1990; Nosaka et al., 1991; Brown et al., 1997; Nosaka and Clarkson, 1997; Foley et al., 1999; Nosaka et al., 2005a; Chen et al., 2007; Howatson et al., 2007; Nikolaidis et al., 2007; Chen et al., 2009; Barroso et al., 2010; Janecki et al., 2011; Muthalib et al., 2011; Meneghel et al., 2014; Lau et al., 2015a; Zourdos et al., 2015; Hinks et al., 2021; Chen et al., 2023) relied on internal/default comparator contrasts. These categories contributed 412 and 260 effect sizes, respectively, and were analyzed separately because internal/default comparator selection may not preserve the original randomization structure and may introduce residual selection bias.

The initial intervention strategies used to prevent EIMD varied across studies (**Supplementary Table S4**). The most frequently implemented intervention was high-intensity eccentric contraction (Ebbeling and Clarkson, 1990; Nosaka et al., 1991; Smith et al., 1994; Brown et al., 1997; Foley et al., 1999; Chen and Hsieh, 2000; Nosaka et al., 2001; Paddon-Jones and Abernethy, 2001; Chen, 2003; Nosaka et al., 2005a; Chen and Nosaka, 2006; Chen et al., 2007; Howatson et al., 2007; Nikolaidis et al., 2007; Chen et al., 2009; Barroso et al., 2010; Chapman et al., 2011; Janecki et al., 2011; Muthalib et al., 2011; Meneghel et al., 2014; Lau et al., 2015a; Hinks et al., 2021; Chen et al., 2023), followed by low-intensity or low-load eccentric contraction (Lavender and Nosaka, 2008; Chen et al., 2012a; Lin et al., 2015; Huang et al., 2019), maximal isometric contraction (Chen et al., 2012b; Chen et al., 2013a; Tseng et al., 2016; Barreto et al., 2019), and high-intensity concentric contraction (Nosaka and Clarkson, 1997). Additionally, three studies (Nosaka and Clarkson, 1997; Tseng et al., 2016; Ho et al., 2021) compared the effects of different first-bout interventions on muscle damage, and one study (Nosaka and Clarkson, 1997) used inclined bench curls, a mixed-contraction form, as the intervention stimulus.

The time intervals between the conditioning and damaging bouts varied across studies, and several studies included multiple interval conditions. The most prevalent interval was within one week (Smith et al., 1994; Chen and Hsieh, 2000; Paddon-Jones and Abernethy, 2001; Chen, 2003; Chen and Nosaka, 2006; Lavender and Nosaka, 2008; Chen et al., 2012b; Zourdos et al., 2015; Barreto et al., 2019; Huang et al., 2019; Ho et al., 2021), followed by 1 to 2 weeks (Nosaka and Clarkson, 1997; Nosaka et al., 2001; Howatson et al., 2007; Barroso et al., 2010; Chapman et al., 2011; Meneghel et al., 2014; Tseng et al., 2016; Chen et al., 2023), 2 to 3 weeks (Brown et al., 1997; Chen et al., 2007; Nikolaidis et al., 2007; Janecki et al., 2011; Chen et al., 2012), and longer than 3 weeks (Foley et al., 1999; Nosaka et al., 2005a; Chen et al., 2009; Muthalib et al., 2011; Lau et al., 2015a; Hinks et al., 2021). Five studies (Brown et al., 1997; Nosaka et al., 2001; Nosaka et al., 2005a; Chen et al., 2009; Barroso et al., 2010) systematically compared the effects of different interval durations on intervention outcomes.

For muscle damage biomarkers, CK (Ebbeling and Clarkson, 1990; Nosaka et al., 1991; Smith et al., 1994; Brown et al., 1997; Nosaka and Clarkson, 1997; Foley et al., 1999; Chen and Hsieh, 2000; Nosaka et al., 2001; Paddon-Jones and Abernethy, 2001; Chen, 2003; Nosaka et al., 2005a; Chen and Nosaka, 2006; Chen et al., 2007; Howatson et al., 2007; Nikolaidis et al., 2007; Lavender and Nosaka, 2008; Chen et al., 2009; Barroso et al., 2010; Chapman et al., 2011; Muthalib et al., 2011; Chen et al., 2012b; Chen et al., 2012a; Chen et al., 2012; Chen et al., 2013a; Meneghel et al., 2014; Lin et al., 2015; Zourdos et al., 2015; Tseng et al., 2016; Huang et al., 2019; Hinks et al., 2021; Ho et al., 2021; Chen et al., 2023) and MB (Nosaka et al., 2001; Chen and Nosaka, 2006; Chen et al., 2007; Chen et al., 2009; Chen et al., 2012b; Chen et al., 2012a; Chen et al., 2012; Chen et al., 2013a; Lin et al., 2015; Tseng et al., 2016; Huang et al., 2019) were the most frequently assessed indicators, with only three studies (Janecki et al., 2011; Lau et al., 2015a; Barreto et al., 2019) not assessing relevant blood markers. DOMS was primarily assessed using visual analog scales (Nosaka and Clarkson, 1997; Nosaka et al., 2001; Chen, 2003; Nosaka et al., 2005a; Chen and Nosaka, 2006; Chen et al., 2007; Howatson et al., 2007; Lavender and Nosaka, 2008; Chen et al., 2009; Barroso et al., 2010; Chapman et al., 2011; Janecki et al., 2011; Muthalib et al., 2011; Chen et al., 2012b; Chen et al., 2012a; Chen et al., 2012; Chen et al., 2013a; Lau et al., 2015a; Lin et al., 2015; Zourdos et al., 2015; Tseng et al., 2016; Barreto et al., 2019; Huang et al., 2019; Hinks et al., 2021; Ho et al., 2021; Chen et al., 2023), while six studies (Ebbeling and Clarkson, 1990; Smith et al., 1994; Foley et al., 1999; Chen and Hsieh, 2000; Nikolaidis et al., 2007; Meneghel et al., 2014) used Likert scales for subjective pain scoring, with higher scores indicating greater perceived soreness. Muscle swelling was assessed using arm circumference (Ebbeling and Clarkson, 1990; Nosaka et al., 1991; Brown et al., 1997; Foley et al., 1999; Chen and Hsieh, 2000; Nosaka et al., 2001; Chen, 2003; Chen and Nosaka, 2006; Chapman et al., 2011; Chen et al., 2012; Lau et al., 2015a; Lin et al., 2015; Tseng et al., 2016; Huang et al., 2019; Hinks et al., 2021; Chen et al., 2023) thigh circumference (Chen et al., 2007; Chen et al., 2012), and ultrasound imaging in various studies (Nosaka et al., 2005a; Chen et al., 2009; Chapman et al., 2011; Muthalib et al., 2011; Chen et al., 2012b; Chen et al., 2012a; Chen et al., 2013a; Lau et al., 2015a; Lin et al., 2015; Tseng et al., 2016; Barreto et al., 2019; Chen et al., 2023). The main functional performance measures included MVC force (Ebbeling and Clarkson, 1990; Nosaka et al., 1991; Smith et al., 1994; Brown et al., 1997; Nosaka and Clarkson, 1997; Chen and Hsieh, 2000; Nosaka et al., 2001; Paddon-Jones and Abernethy, 2001; Chen, 2003; Nosaka et al., 2005a; Chen and Nosaka, 2006; Chen et al., 2007; Howatson et al., 2007; Nikolaidis et al., 2007; Lavender and Nosaka, 2008; Chen et al., 2009; Barroso et al., 2010; Chapman et al., 2011; Janecki et al., 2011; Muthalib et al., 2011; Chen et al., 2012b; Chen et al., 2012a; Chen et al., 2012; Chen et al., 2013a; Lau et al., 2015a; Lin et al., 2015; Zourdos et al., 2015; Tseng et al., 2016; Barreto et al., 2019; Huang et al., 2019; Hinks et al., 2021; Ho et al., 2021; Chen et al., 2023) and peak torque (Nosaka and Clarkson, 1997; Paddon-Jones and Abernethy, 2001; Chen et al., 2007; Howatson et al., 2007; Nikolaidis et al., 2007; Chen et al., 2009; Chapman et al., 2011; Muthalib et al., 2011; Chen et al., 2012b; Chen et al., 2012a; Chen et al., 2012; Chen et al., 2013a; Lau et al., 2015a; Lin et al., 2015; Zourdos et al., 2015; Tseng et al., 2016; Barreto et al., 2019; Hinks et al., 2021; Ho et al., 2021; Chen et al., 2023) of the elbow flexor or knee extensor.

### 3.3 Methodological quality and risk of bias

The Kmet scores of the included studies ranged from 19 to 27, with a mean score of 21.8 (**Supplementary Table S5**), indicating overall moderate to high methodological quality. All included studies satisfied the inclusion criteria, used consistent measurement methods at baseline and on post-intervention days 1–4, recruited participants with relatively similar training backgrounds, applied a standardized RBE protocol, administered damaging exercise at least 24 hours after the conditioning bout, reported at least two EIMD-related outcomes including MVC, DOMS, CK, or muscle circumference, and presented measures of central tendency and dispersion. Among the included studies, 28 were rated as good quality, 7 as excellent quality, and none as low quality.

### 3.4 Overall effects of RBE on EIMD

The three-level meta-analysis revealed significant overall protective effects of RBE across all three outcome measures (**Table 2**). For MVC, the intervention demonstrated a large positive effect (g = 1.18, 95% CI: 0.91–1.45, p < 0.001), indicating substantial preservation of muscle strength. For DOMS, a large negative effect was observed (g = −1.37, 95% CI: −1.70 to −1.05, p < 0.001), reflecting meaningful attenuation of perceived muscle soreness. For CK, a significant negative effect was detected (g = −0.91, 95% CI: −1.33 to −0.50, p < 0.001), suggesting reduced biochemical evidence of muscle damage. Findings from the three-level and two-level models were highly consistent across all three outcomes.

**Table 2:** Overall effect, sensitivity, and heterogeneity analysis of RBE on EIMD.

Outcome	Analysis set	k	g	95% CI	95% PI	p	Q	*I* ^²^	L1%	L2 *χ*^²^/%	L3 *χ*^²^/%
MVC	Before	254	1.18	[0.91, 1.45]	[−0.26, 2.61]	***	502.49***	49.21%			
	After	253	1.17	[0.91, 1.43]	[−0.24, 2.58]	***	487.51***	47.73%	48.40%	~/~	103.99*/51.60%
DOMS	Before	236	−1.37	[−1.70, −1.05]	[−3.20, 0.47]	***	530.90***	55.78%			
	After	234	−1.35	[−1.65, −1.05]	[−3.05, 0.35]	***	490.55***	52.39%	39.81%	2.94/7.29%	76.88***/52.91%
CK	Before	182	−0.91	[−1.33, −0.50]	[−3.24, 1.41]	**	577.26***	71.22%			
	After	179	−0.97	[−1.33, −0.61]	[−2.94, 1.00]	***	478.57***	64.30%	33.40%	7.39*/12.51%	73.27***/54.10%

MVC outlier: Nosaka 2005 (effectsizeID 60); DOMS outliers: Zourdos 2015 (effectsizeIDs 195, 197); CK outliers: Chen 2003 (effectsizeID 53), Chen 2006 (effectsizeIDs 64, 68). Before, full dataset; After, outliers removed. k, number of effect sizes; g, Hedges’ g; CI, confidence interval; PI, prediction interval; Q, Cochran’s Q; *I*^2^, total heterogeneity; L1, Level 1 (sampling); L2, Level 2 (within-study); L3, Level 3 (between-study) variance. *p < 0.05; **p < 0.01; ***p < 0.001. ≈ 0 indicates variance approaching zero.

### 3.5 Sensitivity analyses

Influence diagnostics were conducted to assess whether individual studies exerted disproportionate influence on the pooled estimates (Viechtbauer and Cheung, 2010). Potentially influential studies were identified in outcome-specific analyses for MVC (Nosaka et al., 2005a), DOMS (Zourdos et al., 2015), and CK (Chen, 2003; Chen and Nosaka, 2006). After these studies were excluded, the direction of the pooled effects and the main inferential conclusions were not materially altered. (**Supplementary Figures S1**–**Supplementary Figures S3**).

Design-adjusted sensitivity analyses showed that the pooled estimates were directionally stable when paired-effect variances were recalculated under assumed within-participant correlations of r = 0.30, 0.50, and 0.70. Under the r = 0.50 assumption, pooled effects remained statistically significant for MVC, DOMS, and CK, with detailed estimates reported in **Supplementary Table S6**. In design-stratified models, effects were directionally consistent in both between-participant/control-comparator evidence and within-participant/crossover/repeated-bout evidence. The magnitude of effects differed by design category, particularly for MVC and CK. These findings suggest that the direction of effect was robust to plausible correlation assumptions, whereas effect magnitude remained partly design-dependent, particularly for MVC and CK.

Comparator-stratified analyses showed directionally consistent effects in both explicitly specified control/comparator studies and internal/default comparator contrasts (**Supplementary Table S7**). The larger CK effect observed in internal/default comparator contrasts suggests that comparator definition may influence the estimated magnitude of biochemical outcomes. Therefore, the pooled estimates should be interpreted as intervention-contrast estimates rather than as strictly randomized controlled effects.

### 3.6 Heterogeneity analysis

After removing high-influence studies, the effect sizes for all three outcome measures showed significant heterogeneity (Q test, p < 0.001) (**Table 2**). Log-likelihood ratio tests, in which within-study (level 2) and between-study (level 3) variances components were sequentially constrained, indicated that CK showed significant level 2 variation (χ ^2^ = 7.39, p < 0.05), while MVC and DOMS did not reach significance at this level. All three outcomes showed significant level 3 variation (all p < 0.001), supporting the retention of this hierarchical component.

Stratified *I*^2^ analysis revealed that total variation in CK, DOMS, and MVC was predominantly attributable to between-study differences (54.10%, 52.91%, and 51.60%, respectively), suggesting that methodological variability, sample characteristic, or intervention design represent major sources of heterogeneity. Within-study variation contributed relatively little, with CK showing the largest within-study component (12.51%) and MVC approaching zero. Sampling error accounted for part of the variation in MVC (48.40%) and DOMS (39.81%).

Although MVC and DOMS did not show significant level 2 variation, the three-level model with its nested structure provides a characterization of variation partitioning across hierarchical levels (Harrer et al., 2021).

### 3.7 Time-course of RBE protection

Time-stratified analyses described the temporal pattern of RBE-related protection for each outcome across the sampled recovery period (**Table 3**; **Figure 2**).

**Table 3:** Time-specific pooled protective effects and time-point contrasts.

Outcome	Time	k	g	95% CI	p	Time contrast(h)	Δg (95% CI)	P(Holm)
MVC	24 h	64	0.97	[0.71, 1.22]	< 0.001	72−24	0.501 [0.238, 0.764]	0.0036
MVC	48 h	69	1.12	[0.85, 1.39]	< 0.001	72−48	0.346 [0.131, 0.561]	0.0138
MVC	72 h	61	**1.47**	[1.07, 1.87]	< 0.001	—	—	—
MVC	96 h	60	1.20	[0.86, 1.53]	< 0.001	72−96	0.269 [−0.048, 0.586]	0.2583
DOMS	24 h	62	−1.09	[−1.49, −0.70]	< 0.001	72−24	−0.560 [−0.930, −0.190]	0.0219
DOMS	48 h	65	−1.51	[−1.86, −1.17]	< 0.001	72−48	−0.139 [−0.364, 0.087]	0.5305
DOMS	72 h	60	**−1.65**	[−2.06, −1.25]	< 0.001	—	—	—
DOMS	96 h	49	−1.36	[−1.63, −1.09]	< 0.001	72−96	−0.291 [−0.639, 0.058]	0.3915
CK	24 h	38	−0.28	[−0.90, 0.35]	0.373	—	—	—
CK	48 h	45	−0.66	[−1.17, −0.15]	0.013	48−24	−0.383 [−0.600, −0.167]	0.0038
CK	72 h	50	−1.17	[−1.57, −0.77]	< 0.001	72−24	−0.889 [−1.325, −0.454]	0.0019
CK	96 h	49	**−1.53**	[−1.87, −1.20]	< 0.001	96−24	−1.257 [−1.899, −0.616]	0.0024

k, number of effect sizes; g, Hedges’ g; CI, confidence interval. Δg represents the difference in pooled effects between the first- and second-listed time points. Time-contrast p values are Holm-adjusted across all six pairwise comparisons within each outcome; contrast 95% CIs are unadjusted for multiplicity. Bold values indicate the largest absolute effect size for each outcome.

**Figure 2:**
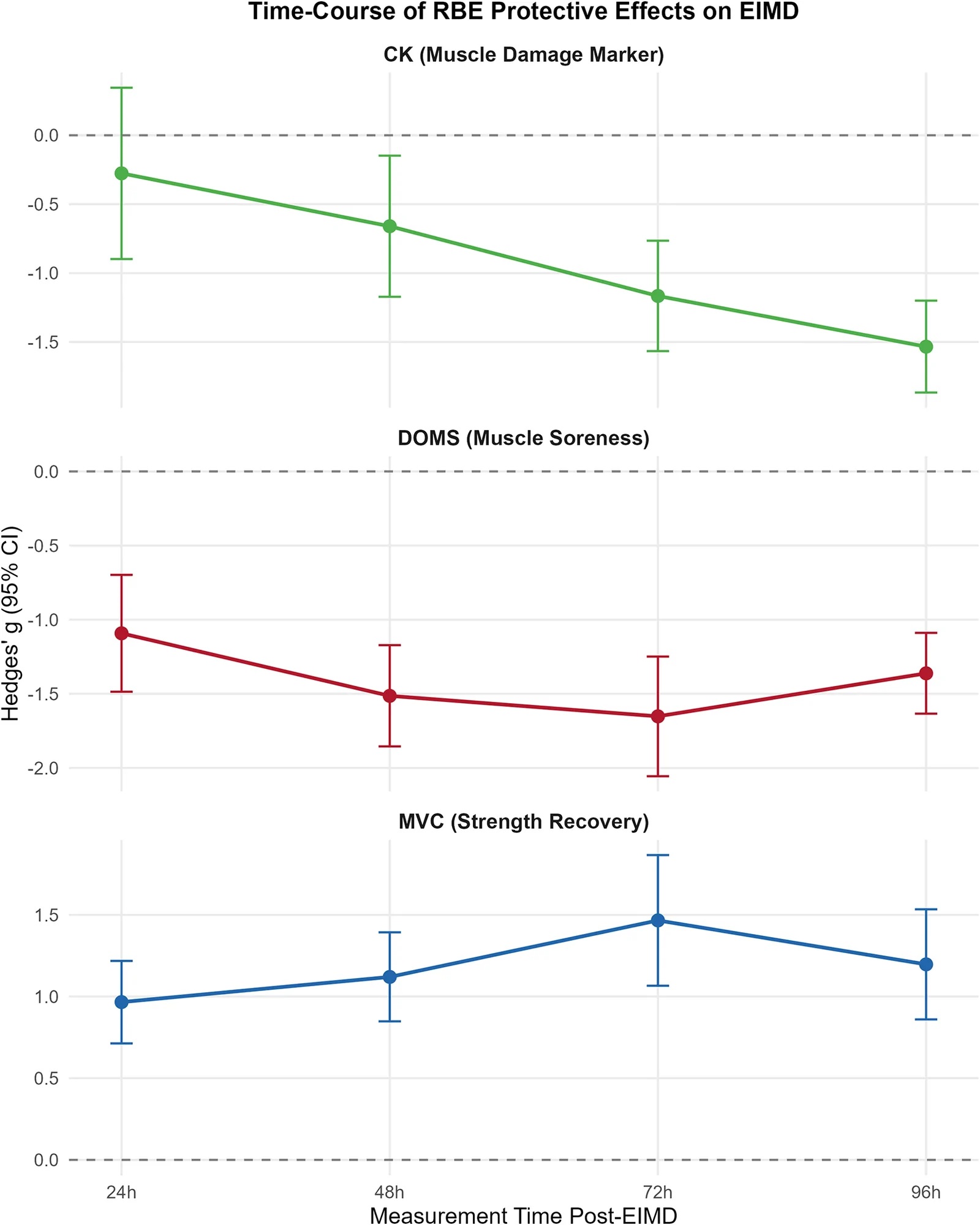
Time-course of RBE protective effects on EIMD.

For MVC, the pooled protective effect was significant at 24 h (g = 0.97, 95% CI: 0.71 to 1.22), largest among the assessed time points at 72 h (g = 1.47, 95% CI: 1.07 to 1.87), and smaller at 96 h (g = 1.20, 95% CI: 0.86 to 1.53). The 72–48-h and 72–96-h contrasts were Δg = 0.346 (95% CI: 0.13 to 0.56; p = 0.014) and Δg = 0.269 (95% CI: −0.05 to 0.59; p = 0.258), respectively. Meta-regression showed a positive linear time trend (β = 0.005, p = 0.002); the quadratic term was not significant (p = 0.130).

Protection against DOMS showed a temporal pattern similar to that for MVC. The pooled protective effect was significant at 24 h (g = −1.09, 95% CI: −1.49 to −0.70) and greater in magnitude at 48 h (g = −1.51). Among the assessed time points, its magnitude was greatest at 72 h (g = −1.65, 95% CI: −2.06 to −1.25), with a smaller magnitude at 96 h (g = −1.36). The 72–48-h and 72–96-h contrasts were Δg = −0.139 (95% CI: −0.36 to 0.09; p = 0.53) and Δg = −0.291 (95% CI: −0.64 to 0.06; p = 0.392), respectively. The quadratic time trend was significant (p = 0.0052), supporting a curvilinear temporal pattern.

In contrast, the magnitude of the pooled CK effect increased progressively across the assessed time points. No significant protective effect was observed at 24 h (g = −0.28, 95% CI: −0.90 to 0.35; p = 0.373). The protective effect was significant at 48 h (g = −0.66, p = 0.013), with greater magnitudes at 72 h (g = −1.17) and 96 h (g = −1.53, 95% CI: −1.87 to −1.20). The 48–24-h and 96–24-h contrasts were Δg = −0.383 (95% CI: −0.60 to −0.17; p = 0.004) and Δg = −1.257 (95% CI: −1.90 to −0.62; p = 0.002), respectively. Meta-regression showed a significant negative linear time trend (β = −0.019 per hour, p = 0.0014).

Analyses restricted to studies reporting all four time points included 24 studies for MVC, 23 for DOMS and 20 for CK. The MVC 72–48-h contrast remained significant (Δg = 0.198, 95% CI: 0.05 to 0.34; p = 0.038), as did the CK 48–24-h contrast (Δg = −0.333, 95% CI: −0.52 to −0.15; p = 0.005). The MVC 72–96-h and DOMS 72–48-h and 72–96-h contrasts remained nonsignificant (all p > 0.05). The quadratic time trend for DOMS remained significant (p = 0.031).

### 3.8 Intervention interval × measurement time

Subgroup analyses stratified by intervention interval and measurement time described the temporal patterns of RBE protection across interval groups (**Table 4**; **Figure 3**).

**Table 4A:** Effect sizes (Hedges’ g) stratified by intervention interval and post-exercise measurement time.

Interval	24 h	48 h	72 h	96 h
Panel A: MVC				
< 1 week	0.91*** (0.592, 1.227) n/k = 14/28	1.18*** (0.815, 1.551) n/k = 15/29	1.46*** (0.966, 1.950) n/k = 13/26	1.21*** (0.846, 1.567) n/k = 12/24
1–2 weeks	0.88* (0.235, 1.528) n/k = 8/12	0.90** (0.381, 1.427) n/k = 10/16	1.28** (0.494, 2.069) n/k = 8/12	1.19** (0.443, 1.929) n/k = 10/16
2–3 weeks	0.43* (0.045, 0.816) n/k = 6/16	0.76 (−0.058, 1.577) n/k = 6/16	0.9 (−0.545, 2.353) n/k = 5/15	0.36 (−0.151, 0.874) n/k = 4/12
> 3 weeks	1.59** (0.782, 2.391) n/k = 6/8	1.52** (0.589, 2.444) n/k = 6/8	1.63* (0.503, 2.760) n/k = 6/8	1.53* (0.395, 2.656) n/k = 6/8
Panel B: DOMS				
< 1 week	−1.24** (−2.044, −0.443) n/k = 14/27	−1.63*** (−2.201, −1.063) n/k = 14/27	−1.80*** (−2.348, −1.254) n/k = 13/26	−1.51*** (−2.136, −0.875) n/k = 10/19
1–2 weeks	−1.02** (−1.650, −0.396) n/k = 10/15	−1.38*** (−2.073, −0.677) n/k = 11/17	−1.50** (−2.439, −0.560) n/k = 10/15	−1.57*** (−2.041, −1.105) n/k = 10/15
2–3 weeks	−0.81* (−1.555, −0.062) n/k = 5/13	−1.33* (−2.303, −0.359) n/k = 5/13	−1.39* (−2.545, −0.241) n/k = 4/12	−0.77 (−2.190, 0.645) n/k = 3/7
> 3 weeks	−1.02** (−1.652, −0.389) n/k = 6/7	−1.53** (−2.233, −0.827) n/k = 7/8	−1.50** (−2.150, −0.845) n/k = 6/7	−0.91** (−1.482, −0.340) n/k = 7/8
Panel C: CK				
< 1 week	0.52 (−0.738, 1.769) n/k = 11/17	−0.07 (−1.142, 1.004) n/k = 12/19	−1.13* (−2.018, −0.249) n/k = 12/22	−1.54*** (−2.145, −0.933) n/k = 11/21
1–2 weeks	−0.86** (−1.319, −0.407) n/k = 7/11	−1.01*** (−1.412, −0.615) n/k = 9/14	−1.22*** (−1.622, −0.821) n/k = 10/15	−1.54*** (−2.107, −0.969) n/k = 11/17
2–3 weeks	−1.03 (−2.850, 0.784) n/k = 1/3	−0.81 (−2.761, 1.151) n/k = 2/4	−0.71 (−2.200, 0.780) n/k = 3/6	−0.11 (−1.672, 1.448) n/k = 2/3
> 3 weeks	−0.72* (−1.348, −0.085) n/k = 5/7	−0.91** (−1.496, −0.324) n/k = 6/8	−1.28** (−1.935, −0.625) n/k = 5/7	−1.66*** (−2.328, −0.983) n/k = 6/8

Values are Hedges’ g (95% CI); n and k denote independent studies and effect sizes, respectively. Values without asterisks indicate non-significant effects (p ≥ 0.05). MVC, maximal voluntary contraction; DOMS, delayed onset muscle soreness; CK, creatine kinase. *p < 0.05; **p < 0.01; ***p < 0.001.

**Figure 3:**
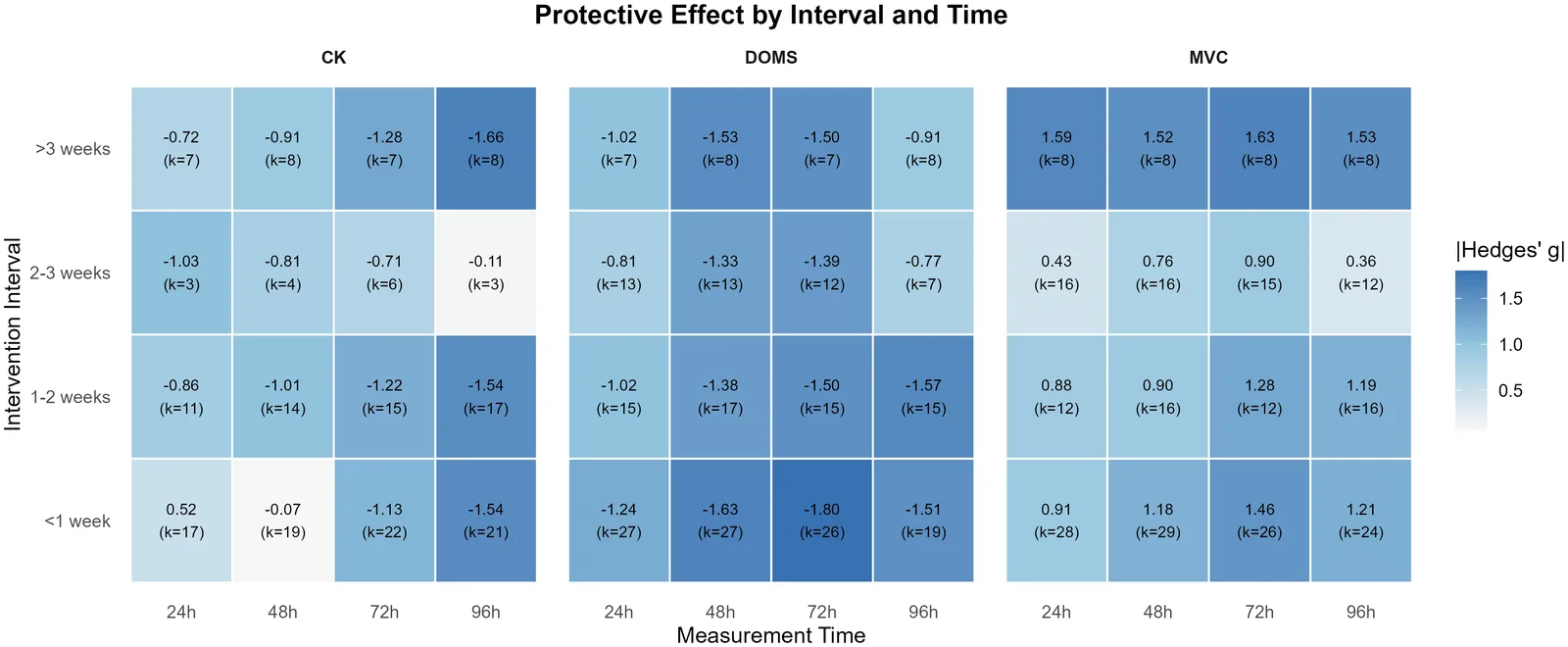
Heatmap of protective effect by interval and time. k, number of effect sizes; colour intensity represents the absolute magnitude of Hedgesʼ g.

With inter-bout intervals of <1 week, pooled protective effects for MVC and DOMS were significant at all four assessed time points (all p < 0.01). For CK, pooled effects were nonsignificant at 24 h (g = 0.52, p = 0.40) and 48 h (g = −0.07, p = 0.89), and significant at 72 h (g = −1.13, p = 0.015) and 96 h (g = −1.54, p < 0.001).

With inter-bout intervals of 1–2 weeks, pooled protective effects were significant across all 12 outcome–time-point combinations (all p < 0.05).

In the 2–3-week subgroup, pooled point estimates ranged from g = 0.36 to 0.90 for MVC and from g = −1.39 to −0.77 for DOMS across the four assessed time points (**Table 4A**). Their magnitudes were numerically smaller than those in the other interval subgroups at each assessment; for CK, this pattern was limited to 72 and 96 h. However, measurement-time-adjusted contrasts with the 1–2-week and >3-week subgroups did not reach statistical significance after Holm correction (**Table 4B**). The corresponding contrasts remained nonsignificant in sensitivity analyses that additionally adjusted for upper- versus lower-limb region or restricted the data to eccentric preconditioning.

**Table 4B:** Measurement-time-adjusted interval contrasts.

Outcome	Interval contrast	Δg	95% CI	p
MVC	2–3 weeks vs 1–2 weeks	−0.382	(−0.869, 0.105)	0.103
MVC	2–3 weeks vs >3 weeks	−1.073	(−2.153, 0.008)	0.103
DOMS	2–3 weeks vs 1–2 weeks	0.731	(−0.790, 2.251)	0.271
DOMS	2–3 weeks vs >3 weeks	0.863	(−0.319, 2.046)	0.271
CK	2–3 weeks vs 1–2 weeks	0.758	(−1.374, 2.889)	0.212
CK	2–3 weeks vs >3 weeks	1.253	(0.077, 2.429)	0.079

Δg is the measurement-time-adjusted effect for the 2–3-week subgroup minus that for the comparison subgroup. p values are Holm-adjusted across two comparisons per outcome; 95% CIs are unadjusted for multiplicity.

With inter-bout intervals of >3 weeks, pooled protective effects were significant across all 12 outcome–time-point combinations (all p < 0.05). Each combination included five to seven independent studies and seven to eight effect sizes (**Table 4**).

### 3.9 Moderator analysis

Body region showed outcome-specific moderation of RBE protection. Compared with upper-limb protocols, lower-limb protocols were associated with smaller protective estimates for MVC (β = −0.716, p = 0.013) and weaker attenuation of DOMS (β = 0.636, 95% CI: 0.035 to 1.237, p = 0.038), whereas no clear moderating effect was observed for CK. Contraction type (eccentric vs. isometric) and contraction intensity (low, moderate, high) did not yield significant overall moderating effects for any of the three outcomes (all p > 0.05). These findings suggest that measurement time remains an important moderator of RBE protection, but muscle region may influence how protection is expressed across functional and perceptual outcomes.

### 3.10 Publication bias

Egger’s regression test indicated significant funnel plot asymmetry for all three outcomes: MVC (t = 17.58, p < 0.0001), DOMS (t = −15.83, p < 0.0001), and CK (t = −3.53, p = 0.0005). Trim-and-fill analysis estimated 52 potentially missing studies for DOMS, 50 for CK, and none for MVC. After correction, effect sizes remained statistically significant for all outcomes. Precision-weighted regression (sda) within the three-level structure suggested small-study effects for MVC (t = 4.20, p < 0.0001), CK (t = −3.25, p = 0.0014), but not for DOMS (t = 0.50, p = 0.617).

All three outcome indicators in the present studies showed evidence of publication bias, with Egger’s regression test reaching statistically significant (p < 0.001). Following trim-and-fill correction, effect sizes remained statistically significant, although several indicators showed reduced magnitude, suggesting that the estimates may be affected by small-study effects or publication bias. For CK, the total sample size was below the GRADE downgrade threshold of 800 participants, resulting in a one-level downgrade due to imprecision. MVC and DOMS were rated as low certainty, while CK was rated as very low certainty (**Table 5**).

**Table 5:** Certainty of evidence for meta-analyzed outcome.

Outcomes	Risk of bias	Indirectness	Publication bias	Inconsistency	Imprecision	Grading of evidence quality
MVC	–	–	↓	–	- (sample size:997)	Low
DOMS	–	–	↓	–	- (sample size:961)	Low
CK	–	–	↓	–	↓ (sample size:765)	Very Low

↓, Evidence downgraded by one level; -, Evidence not downgraded. Sample sizes count each participant once per study and outcome across conditions, comparisons and measurement times. Outcome-specific totals may overlap and should not be summed.

## 4 Discussion

This systematic review and three-level meta-analysis provided the first time-stratified evaluation of the RBE on EIMD. Post-exercise measurement time describes the recovery profile after the damaging bout, whereas the inter-bout interval denotes the time separating conditioning from that bout. Three patterns emerged. First, the protective effect of RBE follows outcome-specific temporal trajectories. MVC and DOMS had their largest observed protective estimates at 72 h, whereas CK had its largest observed protective estimate at 96 h (**Table 3**). Second, the 2–3-week subgroup showed numerically weaker pooled protective effects for MVC and DOMS. Third, temporal variables, including intervention interval and measurement time, may be important moderators of RBE protection (Chen et al., 2012a; Lin et al., 2015; Boyd et al., 2023).

Time-stratified analysis showed that the pooled protective effects for MVC and DOMS were greatest in magnitude at 72 h among the assessed time points, with smaller effects at 96 h. Meta-regression supported a curvilinear temporal pattern for DOMS. Neural adaptations may contribute to MVC protection through changes in motor-unit control (Hyldahl et al., 2017). Changes in biceps brachii motor-unit synchronization have been documented after initial and repeated eccentric bouts (Dartnall et al., 2011). Changes in muscle–tendon mechanics offer a complementary explanation for the preservation of strength and attenuation of soreness. Greater fascicle strain during eccentric contractions has been associated with greater subsequent strength loss (Guilhem et al., 2016), whereas reduced vastus lateralis fascicle elongation during a second bout of eccentric cycling has been associated with attenuated DOMS (Peñailillo et al., 2015). Similarly, smaller increases in biceps brachii myotendinous junction displacement during eccentric exercise after preconditioning have been associated with reduced muscle-damage markers (Lau et al., 2015b; Ho et al., 2021). Together, these observations suggest that reduced local mechanical strain may contribute to functional and perceptual protection, alongside neural adaptations and extracellular matrix (ECM) remodeling (Hyldahl et al., 2017).

CK had its largest observed protective estimate at 96 h, and the estimate at 48 h was more protective than at 24 h. Circulating CK activity also reflects enzyme release and clearance. Remodeling of the extracellular matrix (ECM), including changes in tenascin-C and collagen, may contribute to protection during subsequent exercise (Mackey et al., 2011; Hyldahl et al., 2017). Changes in cytoskeletal proteins and modulation of antioxidant and inflammatory responses may also contribute to RBE-related protection (Markus et al., 2021). The more protective CK estimates at later assessments support including later biochemical measurements alongside earlier assessments in recovery monitoring.

With inter-bout intervals shorter than 1 week, pooled protective effects for MVC and DOMS were significant across all four assessed time points. In this subgroup, CK effects were nonsignificant at 24 and 48 h and significant at 72 and 96 h. Intervals of 1–2 weeks were associated with significant pooled protective effects across all 12 outcome–time-point combinations.

Pooled protective effects for MVC and DOMS were numerically smaller in magnitude in the 2–3-week subgroup. However, measurement-time-adjusted contrasts with the 1–2-week and >3-week subgroups did not reach statistical significance after Holm correction (**Table 4B**). The wide confidence intervals leave substantial uncertainty about the size of these differences. These findings do not establish a distinct period of attenuated protection or identify an optimal inter-bout interval. CK estimates in the 2–3-week subgroup were based on only one to three independent studies at each assessment, limiting their precision. The duration and expression of RBE protection may depend on the combined contributions of neural modulation, altered motor-unit recruitment, improved intermuscular coordination, regulation of inflammatory responses, cytoskeletal stabilization, extracellular matrix remodeling, and changes in muscle–tendon or fascicle behavior (Hyldahl et al., 2017). Previous studies illustrate protocol-specific protection durations. Nosaka et al. (2005b) observed protection against strength loss, soreness and CK responses when maximal eccentric bouts were separated by 12 weeks. Chen et al. (2012a) reported protection with a 2-week interval between low-intensity eccentric conditioning and maximal exercise, with soreness protection also observed at 3 weeks. Differences in exercise model, muscle group, loading intensity, comparator structure and study design may contribute to the interval profiles. In a protocol with 4-week inter-bout intervals, repeated maximal eccentric exercise was associated with attenuated muscle-damage responses in subsequent bouts (Chen et al., 2009). Margaritelis et al. reported that four weeks of intervening concentric training attenuated RBE-related protection, whereas continued eccentric training maintained it (Margaritelis et al., 2015). Intervening training may therefore contribute to between-study heterogeneity and should be considered when interpreting interval-related patterns and developing training recommendations.

Exercise intensity did not significantly modulate the outcome measures, which is consistent with previous research (Chen et al., 2007). Both repeated bouts at identical intensity and low-volume or low-intensity preconditioning can induce protective effects (McHugh and Tetro, 2003; Lavender and Nosaka, 2008; Krentz and Farthing, 2010; Munehiro et al., 2012; Chen et al., 2013b). These interventions may share protective mechanisms, including neural adaptations and extracellular matrix remodeling (Hyldahl and Hubal, 2014; Hyldahl et al., 2017). In multiple studies, Hyldahl et al. (2017) found that low-volume RBE protocols provide effective protection and that severe muscle damage is not a prerequisite for such protection. Similarly, no significant difference was observed between isometric and eccentric contractions, even though eccentric contractions tended to support superior recovery. These findings suggest that temporal variables, including inter-bout interval and measurement time, may be important moderators of RBE protection.

Lower-limb protocols were associated with smaller protective estimates for MVC and weaker attenuation of DOMS, whereas no clear moderating effect was observed for CK. For DOMS, negative effect sizes indicate protection; therefore, the positive lower-limb coefficient represents an effect closer to zero and weaker soreness attenuation relative to upper-limb protocols. Apparent regional differences may reflect variation in exercise protocol, prior eccentric-loading exposure, participant characteristics or outcome assessment. Because these factors may be confounded with body region, the moderator findings remain exploratory and do not establish an intrinsic difference in the capacity of upper- and lower-limb muscles to develop RBE protection.

The present findings extend those of Boyd et al. (2023) in three important ways. First, the time-stratified analysis reveals that overall effect sizes mask substantial temporal variation in protection. Second, the observation of numerically weaker MVC and DOMS effects in the 2–3-week subgroup provides a more nuanced hypothesis regarding interval-related variation in RBE protection than a simple decline with time. Third, joint stratification by interval and measurement time revealed outcome-specific numerical patterns across both temporal dimensions. This supports the use of three-level models that retain time-point-level data rather than collapsing across measurement occasions. In existing RBE literature, traditional two-level meta-analyses typically compare a single preconditioning intervention against a no-preconditioning control at specific time points (Boyd et al., 2023). The three-level meta-analysis employed in this study accounts for effect-size clustering within studies and between-study heterogeneity. Although no significant within-study differences were observed for specific outcomes, retaining this level of variation was considered appropriate given the complexity of the multi-level nested structure (Harrer et al., 2021). Significant between-study heterogeneity indicates that intervention protocols, experimental design, sample characteristics, and measurement timing substantially affect effect magnitudes.

From an applied perspective, these findings suggest that when a high-eccentric-load or otherwise muscle-damaging session is planned, a familiarization or preconditioning bout scheduled within the preceding 1–2 weeks may provide RBE-related protection. For unfamiliar exercises, muscle groups with limited prior eccentric exposure, or athletes returning after a period without eccentric loading, a low-volume re-exposure or gradual loading progression may be considered. For recovery monitoring, MVC and DOMS assessments at 72 h and CK assessments at 96 h, alongside earlier measurements, may help characterize the temporal pattern of RBE-related protection. These recommendations should be individualized according to training status, muscle group, exercise model, and the athlete’s recent eccentric-loading history.

## 5 Limitations

Several limitations should be acknowledged. First, substantial between-study heterogeneity was observed across outcomes, reflecting variation in intervention protocols, outcome measurement, participant characteristics, and study design. Second, between-bout recovery was inconsistently reported in the primary RBE literature, and restoration to the initial baseline was not confirmed for all comparisons. Third, some repeated-measures, crossover-like, or repeated-bout studies did not report the within-participant correlations required for exact paired-effect variance estimation; correlation-adjusted analyses were directionally consistent, but effect magnitudes remained partly design-dependent. Fourth, some studies used internal or default comparator contrasts rather than strictly randomized controlled contrasts, which limits interpretation of the pooled estimates as randomized controlled effects. Finally, small-study effects or publication bias were indicated, GRADE certainty was low for MVC and DOMS and very low for CK, and estimates for intervals longer than three weeks were based on relatively few studies. Most included evidence also came from healthy young adults and relatively controlled exercise models, which limits direct generalization to competitive, multi-joint, or periodized training settings.

## 6 Conclusion

This three-level meta-analysis suggests that RBE-related protective estimates against exercise-induced muscle damage vary across measurement times and intervention interval groups. Functional and perceptual outcomes showed their largest observed protective estimates at 72 h. CK had its largest observed protective estimate at 96 h and a more protective estimate at 48 h than at 24 h. For MVC and DOMS, pooled protective effects were significant at all four assessed time points in the <1-week and 1–2-week subgroups and were numerically smaller in magnitude in the 2–3-week subgroup. Measurement time emerged as an important moderator of RBE protection. These findings identify measurement time and inter-bout interval as complementary temporal dimensions of RBE protection and provide a testable temporal framework for future preconditioning and recovery-monitoring studies.

## Data Availability

The original contributions presented in the study are included in the article/**Supplementary Material**. Further inquiries can be directed to the corresponding author.
