# Supplementary material for: Temporal dynamics of the repeated bout effect on exercise-induced muscle damage: a systematic review and three-level meta-analysis
Source: Front Physiol. 2026 Oct 28;17:1815013. doi: 10.3389/fphys.2026.1815013 (PMC13751514; doi:10.3389/fphys.2026.1815013)
Supplement: Supplementary file 1 [file DataSheet1.pdf]

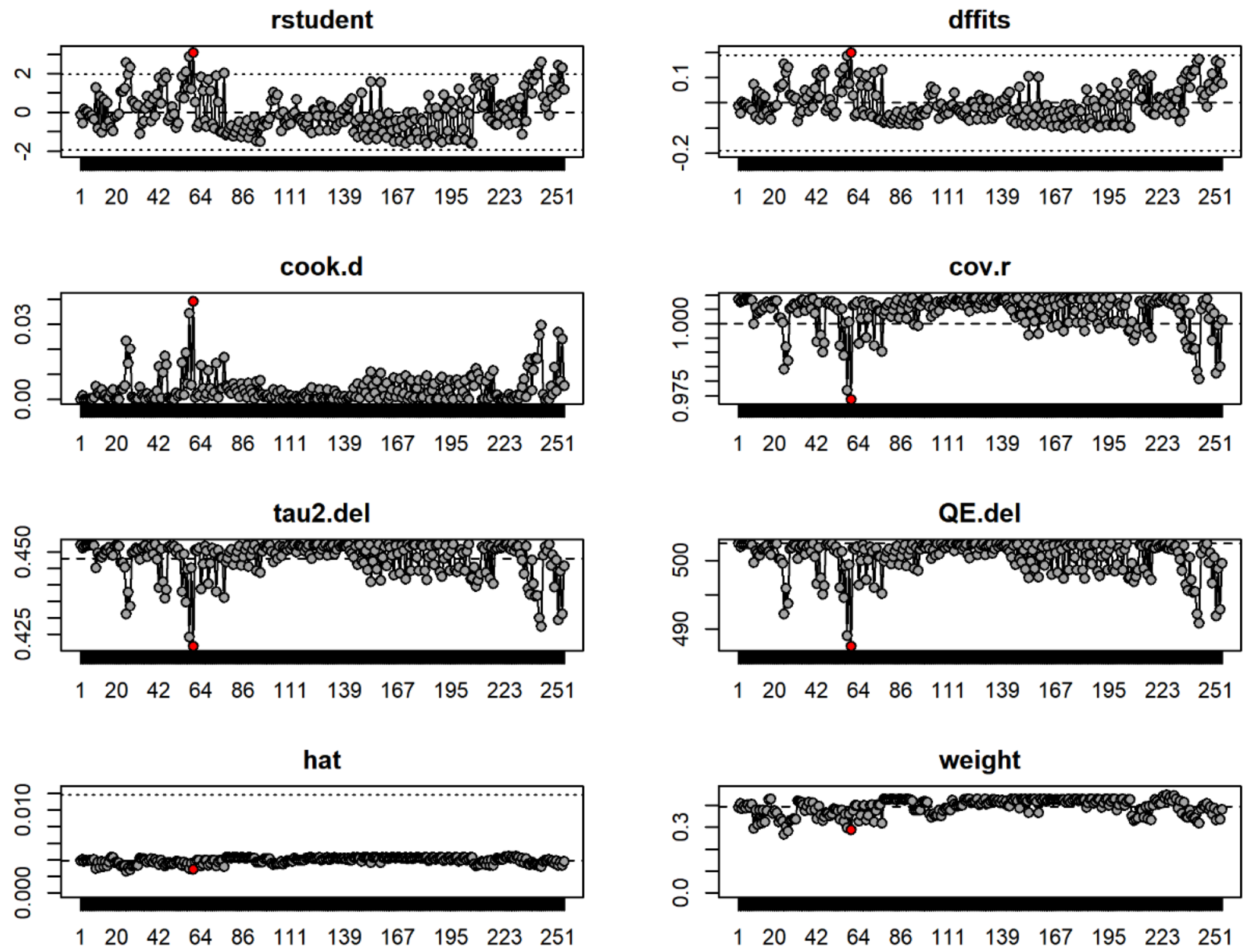

Figure S1. MVC influence.

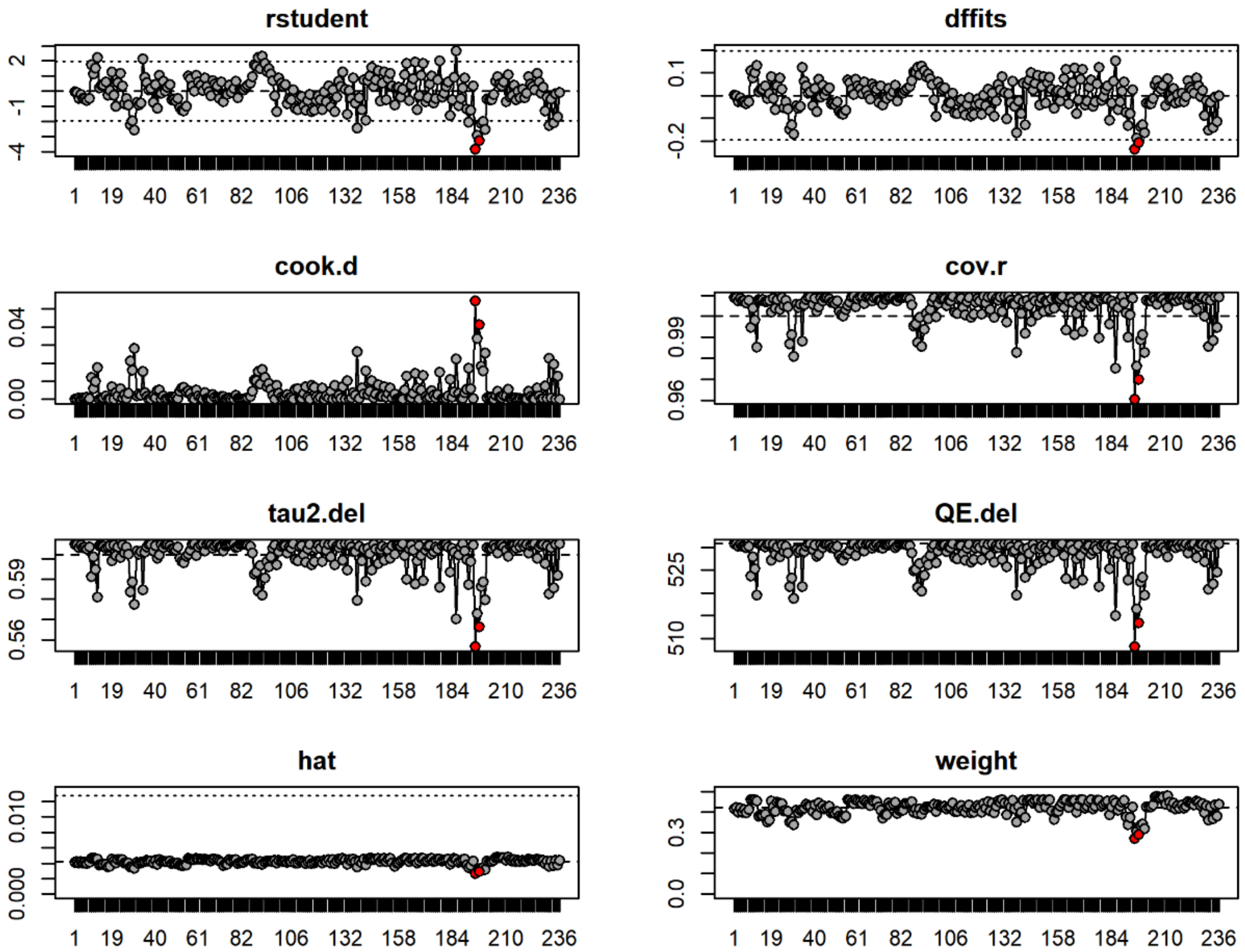

Figure S2. DOMS influence.

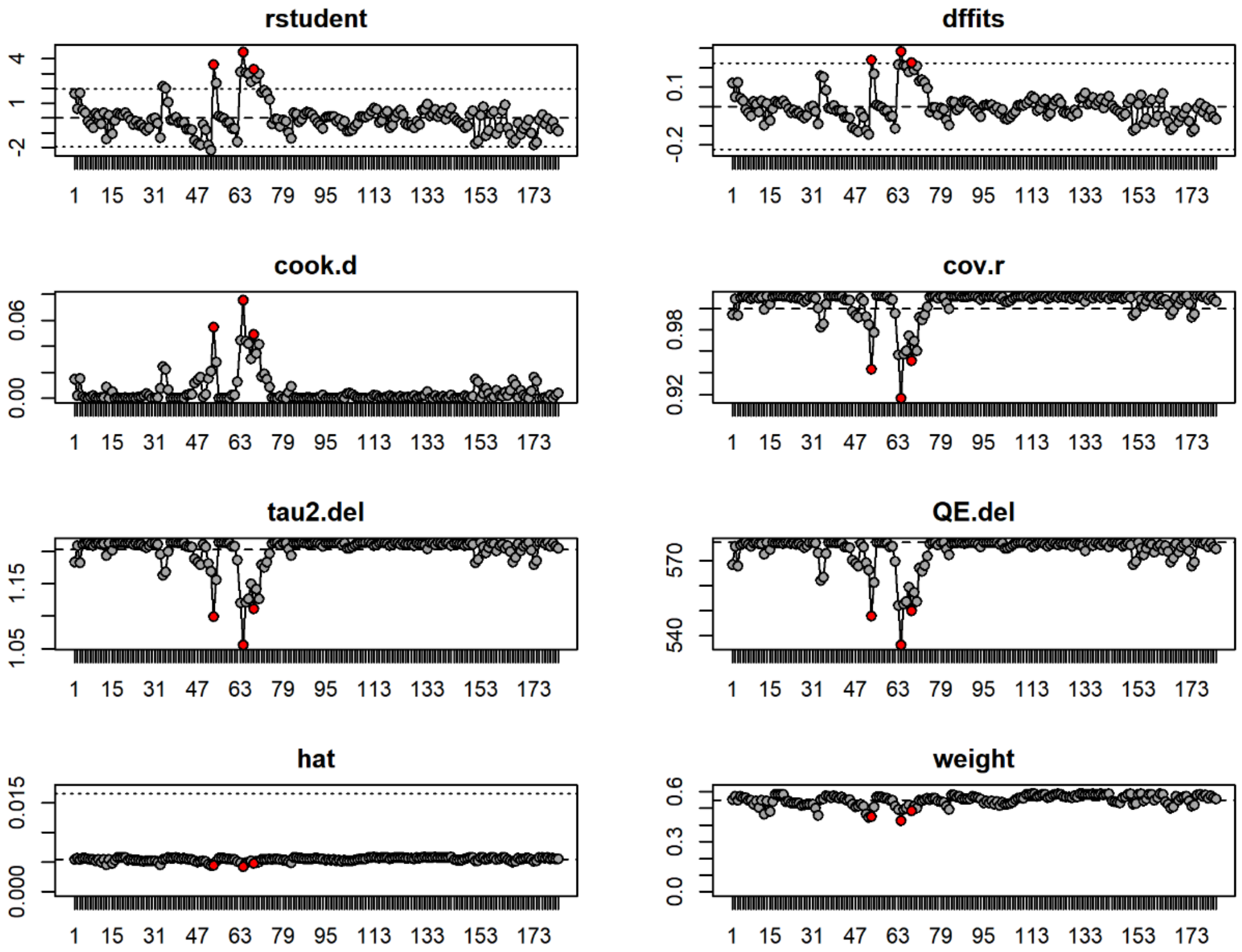

Figure S3. CK influence.

Table S1. Detailed search strategy used in PubMed, Web of Science, Embase, and Scopus.

Database	Search Strategy
PubMed	("repeated bout effect" OR "preconditioning exercise" OR "conditioning contraction*" OR "prior eccentric exercise" OR "initial bout") AND ("eccentric exercise" OR "resistance training" OR "lengthening contraction*" OR "strength training") AND ("muscle damage" OR "exercise-induced muscle damage" OR EIMD OR "muscle soreness" OR DOMS OR "creatine kinase" OR CK OR "myoglobin" OR MB OR "maximal voluntary contraction" OR MVC)
Web of Science	TS=("repeated bout effect" OR "preconditioning exercise" OR "conditioning contraction*" OR "prior eccentric exercise") AND TS=("eccentric exercise" OR "resistance training" OR "lengthening contraction*") AND TS=("muscle damage" OR EIMD OR DOMS OR CK OR MB OR MVC)
Embase	('repeated bout effect' OR 'preconditioning exercise' OR 'conditioning contraction' OR 'prior eccentric exercise') AND ('eccentric exercise' OR 'resistance training' OR 'lengthening contraction') AND ('muscle damage' OR 'exercise induced muscle damage' OR DOMS OR CK OR myoglobin OR MVC)
Scopus	TITLE-ABS-KEY ("repeated bout effect" OR "preconditioning exercise" OR "conditioning contraction*" OR "prior eccentric exercise") AND TITLE-ABS-KEY ("eccentric exercise" OR "resistance training") AND TITLE-ABS-KEY ("muscle damage" OR EIMD OR DOMS OR CK OR myoglobin OR MVC)

Search period: From database inception to June 29, 2025

Filters applied: Human participants; English language; adults

Table S2(A). Characteristics of included studies (n = 35).

Study (first author)	Sample size (n)	Physical characteristics	Comparative groups
Ebbeling 1990	Days 5 group:9<br>Days 14 group:11	The second bout on days 5 and The second bout on days 14<br>Age:20.4 years<br>Height:170 cm<br>Mass:61.1 kg	Experimental group:<br>days 5 and days 14 following the second bout after the muscle-damaging protocol<br>Control group:<br>day 14 following the first bout before the muscle-damaging protocol
Nosaka 1991	6w group:6<br>10w group:8	6w and 10w groups<br>Age:19.2±0.8 years<br>Height:157±5 cm<br>Mass:57.6±7.4 kg	Experimental group:<br>6w and 10w following the second bout after the muscle-damaging protocol<br>Control group:<br>6w and 10w following the first bout before the muscle-damaging protocol
Smith 1994	Experimental group:13<br>No pre-conditioning group:13	Experimental group<br>Age:21.6±0.6 years<br>Height:179.3±2.3 cm<br>Mass:79.1±4.2 kg<br>Body fat:14.7±1.8%<br>No pre-conditioning group:<br>Age:20.6±0.6 years<br>Height:178.8±1.5 cm<br>Mass:77.8±3.6 kg<br>Body fat:14.6±1.3%	Experimental group:<br>performed two bouts of eccentric exercise<br>Control group:<br>No pre-conditioning

Brown 1997	ECC 10 group: 7<br>ECC 30 group: 9<br>ECC 50 group: 8	ECC 10 group:<br>Age:20.5 ± 1.1 years<br>Mass:68.3 ± 9.3 kg<br>ECC 30 group:<br>Age:20.1 ± 0.8 years<br>Mass:63.6 ± 13.9 kg<br>ECC 50 group:<br>Age:22.1 ± 2.4 years<br>Mass:69.3 ± 11.5 kg	Experimental group:<br>ECC 10 and ECC 30 following the second bout after the muscle-damaging protocol<br>Control group:<br>ECC 50 following the first bout before the muscle-damaging protocol
Nosaka 1997	CON-ECC group: 9<br>ECC group: 9	CON-ECC and ECC:<br>Age:23.3 ± 6.7 years<br>Height:159 ± 6<br>Mass:51.4 ± 5.9 kg	Experimental group:<br>CON-ECC<br>Control group:<br>ECC
Foley 1999	group:6	Age:22.3 ±0.6 years<br>Height: 178 ±2cm<br>Mass: 84 ±2kg	Experimental group:<br>Bout 2<br>Control group:<br>Bout 1
Chen 2000	repeated group:12<br>No pre-conditioning group:12	Repeated and control groups<br>Age:22.9±2.3 years<br>Height:173±6.9 cm<br>Mass:68.2±5.1 kg	Experimental group:<br>a groupthat repeated theexercise<br>Control group:<br>No pre-conditioning
Paddon-Jones 2001	ECC group: 7<br>Low volume + ECC group: 8<br>No pre-conditioning group: 8	ECC group, low volume + ECC and no pre-conditioning groups<br>Age:23.9 ± 4.9 years<br>Height:172.6 ± 8.2 cm<br>Mass:67.4 ± 11.6 kg	Experimental group:<br>ECC group and low volume + ECC<br>Control group:<br>No pre-conditioning

Nosaka 2001	2 ECC group: 12<br>6 ECC group: 10<br>24 ECC group 12	2 ECC group, 6 ECC group, 24 ECC groups<br>Age:20.6 (18–27) years<br>Height:172.7 (159–186) cm<br>Mass:61.6 (50–76.3) kg<br>Body fat:15.9% (9.2–26.1%)	Experimental group:<br>2 ECC and 6 ECC following the second bout after the muscle-damaging protocol<br>Control group:<br>24 ECC following the first bout before the muscle-damaging protocol
Chen 2003	EX30 group:8<br>EX70 group:9<br>No pre-conditioning group:9	EX30,EX70 and no pre-conditioning groups<br>Age:20.7±2.5 years<br>Height:175.6±5.6 cm<br>Mass:67.3±6.5 kg<br>Body fat:13.2±6.1%	Experimental group:<br>EX30 and EX70<br>Control group:<br>no pre-conditioning group
Nosaka 2005	S group: 11<br>L group: 11	S and L groups<br>Age:20.3 ± 1.6 years<br>Height:170.1 ± 5.7 cm<br>Mass:61.7 ± 8.7 kg	Experimental group:<br>S group and L group following the second bout after the muscle-damaging protocol<br>Control group:<br>L group performed eccentric exercise at a long starting length for both first (L)
Chen 2006	30 group:10<br>50 group:10<br>70 group:10<br>30-30 group:10<br>30-70 group:10<br>50-50 group:10<br>70-70 group:10	30 group<br>Age:21.5±2.3 years<br>Height:173.5±6.7 cm<br>Mass:63.8±7.1 kg<br>50 group<br>Age:21.3±2.4 years<br>Height:173.1±6.1 cm<br>Mass:63.5±7 kg	Experimental group:<br>30-30,30-70,50-50 and 70-70<br>Control group:<br>30,50 and 70

		70 group<br>Age:20.9±2 years<br>Height:173±6.4 cm<br>Mass:64.1±7.8 kg<br>30-30 group<br>Age:21±2.1 years<br>Height:173.1±6 cm<br>Mass:63.3±7.3 kg<br>30-70 group<br>Age:21.1±2.2 years<br>Height:173.1±6.5 cm<br>Mass:63±7 kg<br>50-50 group<br>Age:21.6±2.3 years<br>Height:173.1±6.2 cm<br>Mass:64±7.7 kg<br>70-70 group<br>Age:21.7±2.4 years<br>Height:172.9±5.8 cm<br>Mass:63.9±7.5 kg	
Nikolaidis 2007	group:12	Age:23±2 years<br>Height:165±2 cm<br>Mass:54±3 kg<br>Body fat:24.9±2%	Experimental group:<br>bout 2<br>Control group:<br>bout 1
Chen 2007	ECC 100% group: 13<br>ECC 80% group: 13	ECC 100%, ECC 80%, ECC 60% and ECC 40% groups:	Experimental group:<br>ECC100%,ECC 80%, ECC 60% and ECC 40% following the second

	ECC 60% group: 13<br>ECC 40% group: 13	Age:20.7 ± 1.9 years<br>Height:173.8 ± 5.0 cm<br>Mass:67.6 ± 7.2 kg	bout after the muscle-damaging protocol Control group:<br>ECC 100% following the first bout before the muscle-damaging protocol
Howatson 2007	ECC 10 group: 8<br>ECC 45 group: 8	ECC 10 and ECC 45 groups:<br>Age:27 ± 5 years<br>Height:180.6 ± 6.9 cm<br>Mass:83.9 ± 10.5 kg	Experimental group:<br>ECC 10 and ECC 45 following the second bout after the muscle-damaging protocol<br>Control group:<br>ECC 45 following the first bout before the muscle-damaging protocol
Lavender 2008	ECC 10%: 9<br>No pre-conditioning group: 9	ECC 10% and no pre-conditioning groups:<br>Age:21.4 ± 2.6 years<br>Height:171.2 ± 5.4 cm<br>Mass:63.7 ± 8.8 kg<br>Body fat:15.8 ± 4.7%	Experimental group: ECC 10%<br>Control group: No pre-conditioning
Chen 2009	group:15	Age: 21.9±1.9 years<br>Height:173.9±6.1 cm<br>Mass:64.9±9.2 kg	Experimental group:<br>Bout 2<br>Control group:<br>Bout 1
Barroso 2010	ECC 60 group:7<br>ECC 180 group:8	ECC60 and ECC180 groups<br>Age:23±3.5 years<br>Height:177.9±8 cm<br>Mass:79.7±9.3 kg	Experimental group:<br>ECC60 and ECC180 following the second bout after the muscle-damaging protocol<br>Control group:<br>ECC60 and ECC180 following the first bout before the muscle-damaging protocol

Chapman 2011	repeated bout group:10<br>No pre-conditioning group group:8	repeated bout and<br>No pre-conditioning groups<br>Age:26.3±4.2 years<br>Height:180±10 cm<br>Mass:79.8±8.2 kg	Experimental group:<br>repeated bout group<br>Control group:<br>No pre-conditioning group
Janecki 2011	group:14	Age:22±2 years<br>Height:180±6 cm<br>Mass:74±8 kg	Experimental group:<br>ECC 2<br>Control group:<br>ECC 1
Muthalib 2011	group:10	Age:29.2±7.1 years<br>Height:175.2±7.6 cm<br>Mass:70.4±9 kg	Experimental group:<br>ECC 2<br>Control group:<br>ECC 1
Chen 2012	Maximum ECC group:13<br>10% ECC group: 13<br>20% ECC group:13<br>90 ISO group: 13<br>20 ISO group: 13	Maximum ECC, 10% ECC, 20% ECC, 90 ISO and 20 ISO groups:<br>Age:20.9 ± 1.8 years<br>Height:174 ± 5.3 cm<br>Mass:67.3 ± 10.6 kg	Experimental group:<br>10% ECC, 20% ECC group, 90 ISO and 20 ISO following the second bout after the muscle-damaging protocol<br>Control group:<br>Maximum ECC following the first bout before the muscle-damaging
Chen 2012	2-day group:13<br>7-day group: 13<br>14-day group:13<br>21-day group: 13<br>No pre-conditioning group: 13	2-day, 7-day, 14-day, 21-day and no pre-conditioning groups:<br>Age:21.3 ± 1.6 years<br>Height:172.3 ± 4.9 cm<br>Mass:71.4 ± 9.9 kg<br>Body fat:25.1 ± 3%	Experimental group:<br>2-day group, 7-day group, 14-day group, 21-day group<br>Control group:<br>No pre-conditioning

Chen 2012	2 MVC-ISO group: 13<br>10 MVC-ISO group: 13<br>No pre-conditioning group: 13	2 MVC-ISO, 10 MVC-ISO and no pre-conditioning groups:<br>Age:22.5 ± 1.7 years<br>Height:172.9 ± 5.7 cm<br>Mass:71.7 ± 9 kg	Experimental group:<br>2 MVC-ISO and 10 MVC-ISO<br>Control group:<br>No pre-conditioning
Chen 2013	0-day group: 13<br>2-day group: 13<br>4-day group:13<br>7-day group: 13<br>No pre-conditioning group: 13	0-day, 2-day, 4-day, 7-day and no pre-conditioning groups:<br>Age:21.8±2.3 years<br>Height:173.6 ± 5.6 cm<br>Mass:69.2±11.1 kg	Experimental group:<br>0-day, 2-day, 4-day and 7-day<br>Control group:<br>No pre-conditioning
Meneghel 2014	group:8	Age:24.4±1.2 years<br>Height:175.2±1.2 cm<br>Mass:75.5±2.3 kg	Experimental group:<br>Bout 2<br>Control group:<br>Bout 1
Lau 2015	group:10	Age:22-28 years<br>Height:170.1±8.6 cm<br>Mass:69.7±14.3 kg	Experimental group:<br>Bout 2<br>Control group:<br>Bout 1
Lin 2015	2-day group: 13<br>1-week group: 13<br>2-week group: 13<br>3-week groups: 13<br>No pre-conditioning group: 13	2-day, 1-week, 2-week and 3-week and no pre-conditioning groups:<br>Age:21.4 ± 1.8 years<br>Height:172.0 ± 5.5 cm<br>Mass:66.3 ± 6.8 kg	Experimental group:<br>2 days, 1 week, 2 weeks and 3 weeks<br>Control group:<br>No pre-conditioning

Zourdos 2015	FE group:10<br>VE group:10	FE group<br>Age:19.7±1.6 years<br>Height:182±7 cm<br>Mass:79.5±10.5 kg<br>VE group<br>Age:21.4±2.4 years<br>Height:179±6 cm<br>Mass:83.2±17.8 kg	Experimental group:<br>FE and VE group following the second bout after the muscle-damaging protocol<br>Control group:<br>FE and VE group following the first bout before the muscle-damaging protocol
Tseng 2016	10 MVC: 13<br>No pre-conditioning group: 13	Experimental and no pre-conditioning groups:<br>Age:21.0 ± 1.2 years<br>Height:171.9 ± 5.6 cm<br>Mass:66.4 ± 8.5 kg	Experimental group:<br>10 MVC<br>Control group:<br>No pre-conditioning group
Huang 2019	10% ECC group: 12<br>No pre-conditioning group: 12	10% ECC and no pre-conditioning groups:<br>Age:21.6 ± 1.8 years<br>Height:172.4 ± 5.9 cm<br>Mass:68.8 ± 11.8 kg	Experimental group:<br>10% ECC<br>Control group:<br>No pre-conditioning
Barreto 2019	ISO + ECC-S group: 16<br>ISO + ECC-F group: 16<br>ECC S group: 16<br>ECC-F group: 16	ISO + ECC-S, ISO + ECC-F, ECC-S and ECC-F groups:<br>Age:21.7 ± 3.1 years<br>Height:174 ± 5.0 cm<br>Mass:74.7 ± 13.6 kg	Experimental group:<br>ISO + ECC-S and ISO + ECC-F<br>Control group:<br>ECC- S and ECC-F
Ho 2021	10%EC group:12<br>2MVIC group:12	10%EC,2MVIC and No pre-conditioning groups<br>Age:21.3±1.5 years	Experimental group:<br>10%EC and 2MVIC

	No pre-conditioning group:12	Height:172±5.4 cm<br>Mass:66.6±8.7 kg	Control group:<br>No pre-conditioning group
Hinks 2021	group:12	Age:23.7±2.3 years<br>Height:177.4±6.6 cm<br>Mass:74.7±13.8 kg	Experimental group:<br>ECC 2<br>Control group:<br>ECC 1
Chen 2023	IL-EF group:13<br>IL-KF group:13	IL-EF and IL-KF groups<br>Age:21±1.8 years<br>Height:171.6±5.6 cm<br>Mass:65.5±7.2 kg	Experimental group:<br>IL-EF and IL-KF following the second bout after the muscle-damaging protocol<br>Control group:<br>IL-EF and IL-KF following the first bout before the muscle-damaging

Table S2(B) Intervention program

study	Intervention Program	duration	Target muscle group	Muscle Injury Treatment Plan	group	outcome measures
Ebbeling 1990	70 eccentric exercise	5 days,14days	the forearm flexors of the non-dominant arm.	Same protocol as the reference bout (ECC1)	Experimental group: days 5 and days 14 following the second bout after the muscle-damaging protocol Control group: day 14 following the first bout before the muscle-damaging protocol	Muscle soreness,Flexed elbow angle,Relaxed elbow angle,Isometric strength and SCK
Nosaka 1991	35 eccentric actions were performed, forcibly extending the forearm from a flexed position (elbow angle ≈ 0.70 rad) to an extended position (elbow angle ≈ 3.05 rad)	6 weeks,10 weeks	the forearm flexors of the non-dominant arm.	Same protocol as the reference bout (ECC1)	Experimental group: 6w and 10w following the second bout after the muscle-damaging protocol Control group: 6w and 10w following the first bout before the muscle-damaging protocol	STR of the forearm flexors, muscle contractures, muscle shortening ability, SOR, and plasma CK activity.
Smith 1994	three sets of 12 repetitions of the eccentric phase of a chest press at an intensity equal to 80% of their previously determined 1-RMc0nc;	2 days	Upper Limb	Same protocol as the reference bout (ECC1)	Experimental group: performed two bouts of eccentric exercise Control group: No pre-conditioning	Delayed onset muscle soreness (DOMS) ratings,CK,Total cholesterol,

Brown 1997	10 (ECC 10), 30 (ECC 30) or 50 (ECC 50) maximal voluntary eccentric contractions of the knee extensors at velocity of 1.05 rad/s	3 weeks	knee extensors	50 maximal voluntary eccentric contractions of the knee extensors of the same leg at velocity of 1.05 rad/s	Experimental group:<br>ECC 10 and ECC 30 following the second bout after the muscle-damaging protocol<br>Control group:<br>ECC 50 following the first bout before the muscle-damaging protocol	Maximal voluntary contraction, CK activity, 20:100 Hz stimulation force, muscle soreness
Nosaka 1997	12 maximal eccentric actions of the elbow extensors with each arm	2 weeks	elbow extensors	100 repetitions of isokinetic concentric contractions of the elbow extensors at an angular velocity of 1.05 rad/s followed by 12 maximal eccentric actions of the elbow extensors with each arm	Experimental group:<br>CON-ECC<br>Control group:<br>ECC	Muscle soreness, isometric force, ROM, CK
Foley 1999	eccentric biceps curls (5 sets of 10 at 110% of the 1-	8 weeks	upper arm	Same protocol as the reference bout (ECC1)	Experimental group:<br>Bout 2	muscle soreness,CK,T2 relaxation times and overall arm swelling

	repetition concentric maximum)				Control group:<br>Bout 1	
Chen 2000	Three sets of 10 repetitions of maximal voluntary isokinetic eccentric exercise were performed by the nondominant elbow flexors on a Cybex 6000 system at an angular velocity of 60°/s	3 days,6 days	non-dominant elbow flexors	Same protocol as the reference bout (ECC1)	Experimental group:<br>a groupthat repeated theexercise<br>Control group:<br>No pre-conditioning	rangeof motionand maximal isometricforce (MIF), muscle soreness index, plasma creatine kinase, and glutamic-oxaloacetate transaminase activities
Paddon-Jones 2001	LV + ECC and ECC group performed 3 maximal eccentric contractions of the elbow flexors at 0.52 and 3.14 rads/s, 3 maximal isometric contractions and 3 maximal concentric contractions at 0.52 and 3.14 rads/s	10 days and 7 days	the elbow flexors	6 sets of 6 maximal eccentric contractions at 0.52 rad/s with 60 s between sets	Experimental group:<br>ECC group and low volume + ECC<br>Control group:<br>No pre-conditioning	Arm girth, elbow angle, muscle soreness, isometric torque, plasma CK, concentric torque, eccentric torque
Nosaka 2001	2, 6 or 24 maximal eccentric contractions of the elbow flexors, performed every 15 s	2 weeks	the elbow flexors	24 eccentric contractions of the elbow flexors every 15 s	Experimental group:<br>2 ECC and 6 ECC following the second bout after the muscle-damaging protocol<br>Control group:<br>24 ECC following the first bout before the muscle-damaging protocol	Maximal isometric force, upper arm circumference, ROM, relaxed elbow joint angle, flexed elbow joint angle, muscle soreness, plasma CK, MB concentration

Chen 2003	30 maximal voluntary isokinetic eccentric contractions (MAX1) on non-dominant elbow flexors at 60° s)1 (1.05 rad s-1)	3 days	non-dominant elbow flexors	Same protocol as the reference bout (ECC1)	Experimental group: EX30 and EX70 Control group: no pre-conditioning group	Upper arm circumference, range of motion, strength, integrated EMG (IEMG), and mean power frequency (MPF),Plasma creatine kinase (CK) activity and muscle soreness
Nosaka 2005	Eccentric action from more elbow flexed position (50°) and ended at 100°, with 2 s duration of eccentric action after a 1-s maximal isometric contraction	4 weeks	elbow	Subject's elbow joint was forcibly extended post 1 s of maximal isometric contraction from a flexed (130°) to an extended (180°) position in 2 s	Experimental group: S group and L group following the second bout after the muscle-damaging protocol Control group: L group performed eccentric exercise at a long starting length for both first (L)	Muscle soreness, isometric force, ROM, CK, echo intensity
Chen 2006	30, 50, or 70 eccentric actions of the elbow flexors of the non-dominant arm using a dumbbell that was set at 80% of each subject's MVC at the elbow angle of 90◦ (1.57 rad)	3 days	the non-dominant arm	Same protocol as the reference bout (ECC1)	Experimental group: 30-30,30-70,50-50 and 70-70 Control group: 30,50 and 70	MVC and active range of motion (ROM),Muscle soreness, upper arm circumference (CIR), serum creatine kinase (CK) activity, and myoglobin (MB) concentration

Nikolaidis 2007	isokinetic exercise session consisting of 75 lengthening knee flexions	3 weeks	knee flexions	Same protocol as the reference bout (ECC1)	Experimental group: bout 2 Control group: bout 1	Isometric torque, range of movement (ROM), delayed-onset muscle soreness (DOMS), creatine kinase (CK), reduced glutathione (GSH), oxidized glutathione (GSSG), thiobarbituric acid–reactive substances (TBARS), protein carbonyls, catalase, uric acid, bilirubin, and total antioxidant capacity (TAC)
Chen 2007	30 eccentric contractions of the elbow flexors with dumbbell set at 40%, 60%, 80% or 100% of participants MVC, repeated every 45 s	2–3 weeks	elbow flexors	30 eccentric contractions of the elbow flexors with dumbbell set at 100% of participants MVC, repeated every 45 s	Experimental group: ECC100%,ECC 80%, ECC 60% and ECC 40% following the second bout after the muscle-damaging protocol Control group: ECC 100% following the first bout before the muscle-damaging protocol	Optimum angle, maximal isometric strength, upper arm circumference, CK activity, MB concentration, ROM, muscle soreness
Howatson 2007	ECC 10 group performed 10 maximal eccentric contractions of the elbow flexors ECC 45 group 3 sets of 15 maximal eccentric	2 weeks	elbow flexors	3 sets of 15 maximal eccentric contractions of the elbow flexors at	Experimental group: ECC 10 and ECC 45 following the second bout after the muscle-damaging protocol Control group: ECC 45 following the first bout	Muscle soreness (mm), percentage change in isometric torque, percentage change in total ROM, log CK

	contractions with 3 min between sets			30°/s with 3 min between sets	before the muscle-damaging protocol	
Lavender 2008	6 sets of 5 eccentric contractions of the elbow flexors, lowering a dumbbell set at 10% of MVC	2 days	elbow flexors	6 sets of 5 eccentric contractions of the elbow flexors, lowering a dumbbell set at 40% of MVC	Experimental group: ECC 10%<br>Control group: No pre-conditioning	Maximal voluntary contraction, ROM, upper arm circumference, plasma CK, muscle soreness
Chen 2009	four bouts of 30 maximal isokinetic eccentric contractions of the elbow flexors	4 weeks	elbow flexors	Same protocol as the reference bout (ECC1)	Experimental group:<br>Bout 2<br>Control group:<br>Bout 1	Maximal voluntary elbow flexion isometric and concentric strength, range of motion at the elbow joint (ROM), upper arm circumference, CK,myoglobin,echo intensity and muscle soreness
Barroso 2010	30 maximal eccentric contractions of the elbow flexors on an isokinetic dynamometer at either 60°·s–1 (Ecc60) or 180°·s–1 (Ecc180).	2 weeks	elbow flexors	Same protocol as the reference bout (ECC1)	Experimental group:<br>ECC60 and ECC180 following the second bout after the muscle-damaging protocol<br>Control group:<br>ECC60 and ECC180 following the first bout before the muscle-damaging protocol	Muscle soreness, ROM, and plasma CK activity,Maximal voluntary isometric contraction strength (MVIC)

Chapman 2011	Maximal isokinetic eccentric exercise was performed at 30°/s, consisting of 35 sets of 6 maximal lengthening contractions (210 total) through a 120° range from 60° (flexed) to 180° (fully extended) at the elbow	2 weeks	nondominant arm	Maximal isokinetic eccentric exercise was performed at 210°/s, consisting of 35 sets of 6 maximal lengthening contractions (210 total) through a 120° range from 60° (flexed) to 180° (fully extended) at the elbow	Experimental group: repeated bout group<br>Control group: No pre-conditioning group	maximal isometric strength, range of motion (ROM), upper-arm circumference, muscle thickness, muscle soreness, serum creatine kinase, and lactate dehydrogenase activities
Janecki 2011	each bout of eccentric exercise that consisted of 30 repetitions of lowering a dumbbell adjusted to 75% of each individual's maximal isometric torque	2-3 weeks	right elbow flexors	Same protocol as the reference bout (ECC1)	Experimental group: ECC 2<br>Control group: ECC 1	Maximal isometric torque, passive muscle stiffness and soreness
Muthalib 2011	10 sets of 6 maximal lengthening contractions of the elbow flexors	4 weeks	elbow flexors	Same protocol as the reference bout (ECC1)	Experimental group: ECC 2	maximal voluntary isometric contraction (MVC) torque, range of motion, plasma creatine kinase

					Control group:<br>ECC 1	activity, muscle soreness, TOImin, and EMGRMS
Chen 2012	ECC groups performed 30 eccentric contractions using dumbbell set to 10%, 20% or 100% MVC. Isometric groups performed 30, 5 s maximal isometric contractions at 90° or 20° elbow flexion	3 weeks	elbow	30 Maximum ECC	Experimental group:<br>10% ECC, 20% ECC group, 90 ISO and 20 ISO following the second bout after the muscle-damaging protocol<br>Control group:<br>Maximum ECC following the first bout before the muscle-damaging	MVC torque, flexed elbow joint angle, ROM, upper arm circumference, CK activity, MB concentration, muscle soreness
Chen 2012	5 sets of six ECC of the elbow flexors set at 10% of MVC, with dumbbells	2 days, 1 week, 2 weeks or 3 weeks	elbow flexors	5 sets of 6 maximal eccentric contractions at angular velocity of 30°/s, 2 min between sets	Experimental group:<br>2-day group, 7-day group, 14-day group, 21-day group<br>Control group:<br>No pre-conditioning	Elbow flexor MVC, optimum angle, upper arm circumference, ROM, muscle soreness, plasma CK, MB concentration, ultrasound echo intensity
Chen 2012	2 or 10 maximal isometric contractions of the elbow flexors, elbow joint angle of 20°	2 days	elbow flexors	5 sets of 6 maximal eccentric contractions of the elbow flexors, angular velocity 90°/s, 2 min between sets	Experimental group:<br>2 MVC-ISO and 10 MVC-ISO<br>Control group:<br>No pre-conditioning	Peak torque, upper arm circumference, ROM, MVC peak torque, plasma CK, plasma MB, ultrasound echo intensity, muscle soreness

Chen 2013	2 maximal voluntary isometric contractions of the elbow flexors, elbow angle 20°	0 days, 2 days, 4 days, 7 days	elbow flexors	5 sets of 6 maximal voluntary eccentric contractions at angular velocity of 90°/s, 2 min between sets	Experimental group: 0-day, 2-day, 4-day and 7-day Control group: No pre-conditioning	MVC-CON, optimum angle, upper arm circumference, muscle soreness, plasma CK, MB concentration, flexed elbow joint angle and extended elbow joint angle, ROM, ultrasound echo intensity
Meneghel 2014	4 sets of 8 eccentric actions (3 seconds for each repetition) at 70% of eccentric 1 repetition maximum (1RM) load in a bench press exercise with 2 minutes of rest between sets	2 weeks	Upper Limb	Same protocol as the reference bout (ECC1)	Experimental group: Bout 2 Control group: Bout 1	Bench press 1RM, delayed onset muscle soreness (DOMS) assessed by a 6-point Likert scale, serum creatine kinase (CK) activity, and plasma prostaglandin E2 concentration (PGE2)
Lau 2015	ten sets of six maximal isokinetic eccentric contractions of the elbow flexors with the same arm	4 weeks	elbow flexors	Same protocol as the reference bout (ECC1)	Experimental group: Bout 2 Control group: Bout 1	Maximal voluntary isometric contraction torque, range of motion, muscle soreness assessed by a visual analogue scale (VAS) and pressure pain threshold
Lin 2015	Knee flexors 3 sets of 10, knee extensors 6 sets of 10 low intensity eccentric contractions	2 days, 1 week, 2 weeks or 3 weeks	knee extensors	Knee flexors 3 sets of 10, knee extensors 6 sets of 10 of maximal	Experimental group: 2 days, 1 week, 2 weeks and 3 weeks Control group: No pre-conditioning	MVC-CON torque, peak torque, ROM, plasma CK, MB concentration, muscle soreness, ultrasound echo intensity

				eccentric contractions		
Zourdos 2015	5 sets of 6 repetitions (load = 45–55% MIEFS) of the incline dumbbell curl exercise using the nondominant arm with 3 minutes rest between sets.	1 weeks	Upper Limb	FE repeated the exercise protocol used in session 1 (i.e., incline to incline), whereas VE performed the dumbbell preacher curl exercise with the nondominant arm using the same set, repetition, and load configuration as the first session (i.e., incline to preacher).	Experimental group:<br>FE and VE group following the second bout after the muscle-damaging protocol<br>Control group:<br>FE and VE group following the first bout before the muscle-damaging protocol	maximal voluntary isometric and isokinetic torque, range of motion, muscle soreness, and serum creatine kinase
Tseng 2016	6 sets of 10 maximal isometric contractions of the knee extensors at 90°	2 weeks	knee extensors	6 sets of 10 maximal eccentric	Experimental group:<br>10 MVC	MVC-CON, thigh girth, muscle soreness, plasma CK, MB

	knee joint angle, 2 min between sets			contractions of the knee extensors, angular velocity of 30°/s. 2 min between sets	Control group: No pre-conditioning group	concentration, ROM, ultrasound echo intensity
Huang 2019	5 sets of 10 eccentric contractions at 10% MVC	2 days	elbow flexors, knee flexors and knee extensors	5 sets of 10 eccentric contractions at 80% MVC	Experimental group: 10% ECC Control group: No pre-conditioning	MVC strength, muscle soreness, plasma CK, MB concentration
Barreto 2019	10 MVC with elbow flexed at 20°	2 days	elbow	ECC-S group performed 30 maximal eccentric contractions at 60°/seconds	Experimental group: ISO + ECC-S and ISO + ECC-F Control group: ECC- S and ECC-F	Maximal isokinetic torque concentric torque, muscle soreness, muscle thickness
Ho 2021	30 low-intensity (10%) eccentric contractions (10%EC) or two maximal voluntary isometric contractions at a long muscle length (2MVIC)	2 weeks	elbow flexors	maximal eccentric exercise of the elbow flexors consisting of five sets of six maximal eccentric	Experimental group: 10%EC and 2MVIC Control group: No pre-conditioning group	Biceps brachii distal myotendinous junction (MTJ) displacement,maximal voluntary concentric contraction (MVC-CON) torque, (ROM), muscle soreness, and plasma CK activity

				contractions (MaxEC)		
Hinks 2021	5 sets of 30 maximal eccentric elbow flexions	4 weeks	elbow flexions	Same protocol as the reference bout (ECC1)	Experimental group: ECC 2<br>Control group: ECC 1	CK,muscle soreness,Maximum voluntary isometric contraction
Chen 2023	30 maximal eccentric contractions of the EF;60 maximal eccentric contractions of the KF(30°/s)	2 weeks	elbow flexors (EF) and knee flexors (KF)	Same protocol as the reference bout (ECC1)	Experimental group: IL-EF and IL-KF following the second bout after the muscle-damaging protocol<br>Control group: IL-EF and IL-KF following the first bout before the muscle-damaging	muscle soreness, range of motion (ROM), upper or lower limb circumference (CIR), muscle hardness, echo intensity of ultrasound transverse images (EI), position sense (PS), joint reaction angle to release (JRA), and performed submaximal (30%, 50%, 80%) and maximal voluntary concentric contractions (MVC) of the EF or KF.

Table S3. Participant characteristics.

Variable	RBE/preconditioning conditions	Comparator conditions
Mean age (years)	21.5	22.3
Mean height (cm)	173	173.2
Mean body mass (kg)	67.3	69.1
Training status	Untrained or recreationally active	Similar baseline

These columns describe exercise conditions. In within-participant designs, the same individuals may be represented in both columns.

Table S4. Summary of intervention characteristics.

**Category**	**Description**
Most common contraction	High-intensity eccentric
Low-load protocols	10–40% MVC
Isometric preconditioning	2 maximal contractions
Concentric preconditioning	Limited (n = 1–2)
Most common muscle group	Elbow flexors
Measurement time points	24, 48, 72, 96 h
Most frequent interval	<1 week

Table S5. Methodological quality (Kmet criteria).

Study Quality Category	Number of Studies
Excellent (>23)	7
Good (>18)	28
Fair	0
Poor	0
Mean Kmet Score	21.8

Table S6. Design-adjusted and design-stratified sensitivity analyses.

Outcome	Analysis	k	Studies	Hedges' g	95% CI	tau^2	Interpretation
MVC	Overall, all rows treated with independent-group variance	254	32	1.01	[0.90, 1.12]	0.58	Direction unchanged across variance assumptions.
MVC	Overall, within-participant variance adjusted with r = 0.30	254	32	1.02	[0.91, 1.13]	0.59	Direction unchanged across variance assumptions.
MVC	Overall, within-participant variance adjusted with r = 0.50	254	32	1.02	[0.91, 1.13]	0.60	Direction unchanged across variance assumptions.
MVC	Overall, within-participant variance adjusted with r = 0.70	254	32	1.02	[0.91, 1.13]	0.61	Direction unchanged across variance assumptions.
MVC	Between-participant/control-	185	20	0.94	[0.81, 1.07]	0.62	Same direction; magnitude differs by

Outcome	Analysis	k	Studies	Hedges' g	95% CI	tau^2	Interpretation
	comparator evidence, r = 0.50 model						design category.
MVC	Within-participant/crossover/repeated-bout evidence, r = 0.50 model	69	14	1.23	[1.04, 1.42]	0.50	Same direction; magnitude differs by design category.
DOMS	Overall, all rows treated with independent-group variance	236	32	-1.34	[-1.46, -1.22]	0.71	Direction unchanged across variance assumptions.
DOMS	Overall, within-participant variance adjusted with r = 0.30	236	32	-1.34	[-1.46, -1.21]	0.73	Direction unchanged across variance assumptions.
DOMS	Overall, within-participant variance adjusted with r = 0.50	236	32	-1.34	[-1.46, -1.21]	0.75	Direction unchanged across variance assumptions.

Outcome	Analysis	k	Studies	Hedges' g	95% CI	tau^2	Interpretation
DOMS	Overall, within-participant variance adjusted with r = 0.70	236	32	-1.34	[-1.46, -1.21]	0.77	Direction unchanged across variance assumptions.
DOMS	Between-participant/control-comparator evidence, r = 0.50 model	168	18	-1.31	[-1.45, -1.18]	0.63	Same direction; magnitude differs by design category.
DOMS	Within-participant/crossover/repeated-bout evidence, r = 0.50 model	68	15	-1.41	[-1.68, -1.14]	1.12	Same direction; magnitude differs by design category.
CK	Overall, all rows treated with independent-group variance	182	28	-0.78	[-0.96, -0.61]	1.21	Direction unchanged across variance assumptions.
CK	Overall, within-participant variance	182	28	-0.79	[-0.96, -0.61]	1.20	Direction unchanged across variance

Outcome	Analysis	k	Studies	Hedges' g	95% CI	tau^2	Interpretation
	adjusted with r = 0.30						assumptions.
CK	Overall, within-participant variance adjusted with r = 0.50	182	28	-0.79	[-0.96, -0.62]	1.20	Direction unchanged across variance assumptions.
CK	Overall, within-participant variance adjusted with r = 0.70	182	28	-0.79	[-0.96, -0.62]	1.19	Direction unchanged across variance assumptions.
CK	Between-participant/control-comparator evidence, r = 0.50 model	115	16	-0.65	[-0.91, -0.38]	1.88	Same direction; magnitude differs by design category.
CK	Within-participant/crossover/repeated-bout evidence, r = 0.50 model	67	14	-0.97	[-1.13, -0.81]	0.29	Same direction; magnitude differs by design category.

Table S7. Comparator-stratified sensitivity analyses.

Outcome	Comparator category	k	Studies	Hedges' g	95% CI	P	tau^2	I^2	Interpretation
MVC	Explicitly specified control/comparator	159	17	0.92	[0.79, 1.05]	<0.001	0.55	72.8%	Direction unchanged; magnitude differs by comparator, clearest for CK.
MVC	Internal/default comparator	95	15	1.23	[1.03, 1.43]	<0.001	0.72	73.5%	Direction unchanged; magnitude differs by comparator, clearest for CK.
DOMS	Explicitly specified control/comparator	148	16	-1.34	[-1.50, -1.19]	<0.001	0.72	77.6%	Direction unchanged; magnitude differs by comparator, clearest for CK.
DOMS	Internal/default comparator	88	16	-1.38	[-1.60, -1.17]	<0.001	0.83	73.8%	Direction unchanged; magnitude differs by comparator, clearest for CK.
CK	Explicitly specified control/comparator	105	14	-0.58	[-0.86, -0.29]	<0.001	2.04	86.6%	Direction unchanged; magnitude differs by

Outcome	Comparator category	k	Studies	Hedges' g	95% CI	P	tau^2	I^2	Interpretation
									comparator, clearest for CK.
CK	Internal/default comparator	77	14	-1.07	[-1.22, -0.91]	<0.001	0.24	50.7%	Direction unchanged; magnitude differs by comparator, clearest for CK.
